# A novel tail fiber protein triggers phage DNA ejection by recognizing lipopolysaccharides of K54 hypervirulent *Klebsiella pneumoniae*

**DOI:** 10.1128/spectrum.02171-25

**Published:** 2025-11-10

**Authors:** Ming Yin, Li Cao, Yu Fu, Yanjun Lu, Yi Yan, Lvxin Qian, Li Xiang, Tiejun Zhou, Huan Chen, Ying Li, Luhua Zhang

**Affiliations:** 1 The School of Basic Medical Sciences, Southwest Medical University 74647, Luzhou, Sichuan, China; 2 Department of Pathology, the Affiliated Hospital of Southwest Medical University 556508, Luzhou, Sichuan, China; Institute of Microbiology, Chinese Academy of Sciences, Beijing, China

**Keywords:** hypervirulent *K. pneumoniae*, CPS, LPS, phage adsorption, DNA ejection, tail fiber protein

## Abstract

Bacteriophage (phage) therapy is a promising alternative antibacterial approach for treating infectious diseases by hv*Kp*. However, there has been a lack of studies on the host recognition and infection initiation of hv*Kp* phages. In this work, we found that both capsular polysaccharide (CPS) and lipopolysaccharide (LPS) of strain SCNJ1 are essential for phage infection, and mutations in genes associated with the CPS or LPS biosynthesis pathway conferred phage resistance. These CPS-related mutants, but not LPS-related mutants, showed virulence attenuation and significantly reduced colonization in mice. We demonstrate that CPS serves as the primary receptor for phage adsorption and can be cleaved by phage depolymerase Dep-Y. Phage DNA ejection was specifically triggered by purified LPS, rendering LPS a secondary receptor during the phage infection process. Furthermore, mutant phages capable of infecting the LPS-related mutant strain were isolated, leading to the identification of a novel tail fiber protein named TFP_Y, which can interact with LPS to initiate phage genome ejection. In conclusion, we propose a molecular model for phage infection in the hv*Kp* strain SCNJ1. In this model, the phage utilizes both the CPS and LPS as receptors to initiate an infection in hv*Kp*: the phage attaches to the bacterial cell surface; depolymerase Dep-Y degrades the host CPS to expose the LPS; and TFP_Y interacts with LPS, leading to the ejection of phage genome DNA. These findings deepen our understanding of phage-hvKp strain interactions during phage infection, which is crucial for developing phage-based therapeutic strategies.

**IMPORTANCE:**

Phage infection begins with the adsorption of the phage particle to the bacterial cell surface, followed by the injection of the phage genome into the cell. In the encapsulated hypervirulent *K. pneumoniae* (hv*Kp*), the capsular polysaccharide (CPS) surrounding the bacterium was assumed to be the adsorption receptor of phage infection. Lipopolysaccharide (LPS) is a common phage receptor in many bacteria. Whether and how CPS and LPS act as receptors simultaneously to initiate the phage infection in hv*Kp* remains unknown. Here, we demonstrated that bacterial CPS and LPS are both critical determinants for phage infection in hv*Kp* strain SCNJ1, and mutations in either component confer complete resistance. We revealed that CPS is the primary receptor for phage adsorption, whereas LPS is the secondary receptor for phage injection. Furthermore, we identified a novel tail fiber protein TFP_Y that recognizes host LPS to start phage genome DNA release.

## INTRODUCTION

Bacterial antimicrobial resistance has emerged as one of the leading public health threats of the 21st century, and the *Review on Antimicrobial Resistance* estimated that antimicrobial resistance could cause 10 million deaths per year by 2050 (1). *Klebsiella pneumoniae* is an opportunistic gram-negative encapsulated bacterium that is included in the most problematic ESKAPE group, which are well-known, highly virulent, and antimicrobial-resistant clinical pathogens (2). Hypervirulent *K. pneumoniae* (hv*Kp*), which often produces more capsule polysaccharide (CPS) than classic *K. pneumoniae* isolates (referred to as hyper-capsule), is an evolving pathotype that is associated with high morbidity and mortality (3). It can infect both immunocompromised and healthy individuals of any age and can infect nearly every site of the body, causing infectious syndromes such as hepatic abscess, pneumonia, endophthalmitis, and meningitis (4). hv*Kp* strains are becoming increasingly resistant to antimicrobials, and notably, the prevalence of carbapenem-resistant hv*Kp* poses a new challenge to public health in China (5). Bacteriophages (phages) are a type of virus that specifically infect or kill, in the case of lytic phages, the bacterial host, and they can be used to treat bacterial infections, especially those caused by drug-resistant pathogens (6). Phage therapy shows great potential in the treatment of *K. pneumoniae* infections, as demonstrated in mouse models and clinical trials (7–10). However, bacteria can readily develop resistance to phage during the treatment process (7, 11–15). Laboratory characterization of the resistance mechanisms of bacteria to phage can greatly benefit the clinical application of phage therapy and help us clarify the structural and molecular mechanisms of phage-host interactions.

Phage adsorption to host receptors is the initial step of infection (16–18). If receptors on the bacterial cell surface become inaccessible or non-complementary to the phage receptor-binding protein (RBP), phage resistance arises (19–21). Most known phages utilize bacterial CPS, components of the bacterial cell wall (e.g., lipopolysaccharide [LPS]), appendages (e.g., pili and flagella), or outer membrane proteins (e.g., OmpA) as receptors (16, 22, 23). In *K. pneumoniae*, the mutation in genes located within the capsule biosynthesis locus, which can lead to the loss or alteration of CPS, is the most common mechanism of phage resistance (13, 24, 25). It is documented that the mutated CPS of *K. pneumoniae* prevents phage attachment and enables the bacteria to evade phage infection (14, 17). In addition, studies showed that the mutations in LPS conferred phage resistance (7, 12, 14), demonstrating that LPS is also essential for efficient phage-host infection. However, the impact of altered LPS on phage adsorption efficiency is contradictory in these studies. It is known that LPS is a common phage adsorption receptor in many gram-negative bacteria, such as *Pseudomonas aeruginosa* (26), *Salmonella spp*. (27), and *Escherichia coli* (28). For the nonmucoid (non-capsulated) *K. pneumoniae* strain A2312NM, the LPS was the reversible adsorption receptor of phage ФNJS1 (29). However, in the hyper-capsulated *K. pneumoniae* strain hvKpP3, the LPS mutation caused phage resistance but did not change the phage adsorption efficiency (12, 14). The roles of LPS of the encapsulated *K. pneumoniae* during the phage infection process are still unclear.

During the phage infection process, the initial reversible attachment of phage to a receptor on the surface of a bacterial cell is followed by irreversible binding and genome ejection into the host cytoplasm (11, 30). The reversible and irreversible receptors can be the same or different. The *E. coli* phage T4, the paradigm of the *Myoviridae* family, utilizes a two-step process to first attach to the host LPS and then bind to the outer membrane protein OmpC (31). A similar mechanism has also been reported in *Shigella flexneri* phage Sf6 (23, 32). For the encapsulated bacteria, their CPS not only can block access to cell wall receptors but also may function as the primary receptor for phage adsorption (18, 20, 33). In the latter case, cell wall components may act as secondary receptors for phage irreversible binding. In *E. coli* strain DE058, CPS is involved in the initial phage adsorption, and LPS mediates the subsequent irreversible binding (18). In the K2 hyper-capsulated *K. pneumoniae* strain B5055, phages NPat and BMac recognize the host via two essential steps, with CPS acting as the primary binding receptor and OmpK36 as the secondary one (17). In *K. pneumoniae*, especially the hyper-capsulated strains, whether LPS and CPS act as receptors simultaneously to initiate the phage infection has remained unknown.

The interaction between phage and its host bacteria begins with the recognition and binding to specific cell-surface structures by RBPs, such as the tail fibers or tail spike proteins located at the tail of the phage particle (33–35). The gp63.1 of *podovirus* phage G7C binds to and deacetylates the LPS O-antigen of *E. coli* strain 4 s to initiate the phage infection (28). The *siphovirus* 9NA and *podovirus* P22 interact with *S. typhimurium* LPS with their tail spike proteins, committing phage DNA ejection (36). In *K. pneumoniae*, many phages encode RBPs with depolymerase activity that specifically degrade the CPS surrounding the bacterial surface, permitting phage irreversible binding to the cell wall (37–39). However, the phage RBP that is in contact with the LPS of *K. pneumoniae* remains to be investigated.

In this study, we begin with the exploration of genetic mechanisms underlying resistance acquisition during phage therapy using the *myovirus* phage vB_KpnM_SCNJ1-Y targeting the hyper-capsulated hv*Kp* strain SCNJ1. We revealed that phage resistance can emerge via either CPS or LPS mutations, supporting that both CPS and LPS function as receptors for phage binding. We further demonstrate that CPS is essential for phage adsorption, and LPS provides a second step of receptor binding for the trigger of phage DNA ejection. Furthermore, we identified a phage tail fiber protein, TFP_Y, as the RBP of host LPS. Protein TFP_Y may interact with LPS to initiate phage genome delivery into its host. This study provides a framework for phage-host interaction during phage attachment and genome injection in hv*Kp*, which has notable implications for the development and application of phage therapies in the treatment of hv*Kp* infections.

## RESULTS

### Selection of phage-resistant clones in response to phage treatment

**Fig 1:**
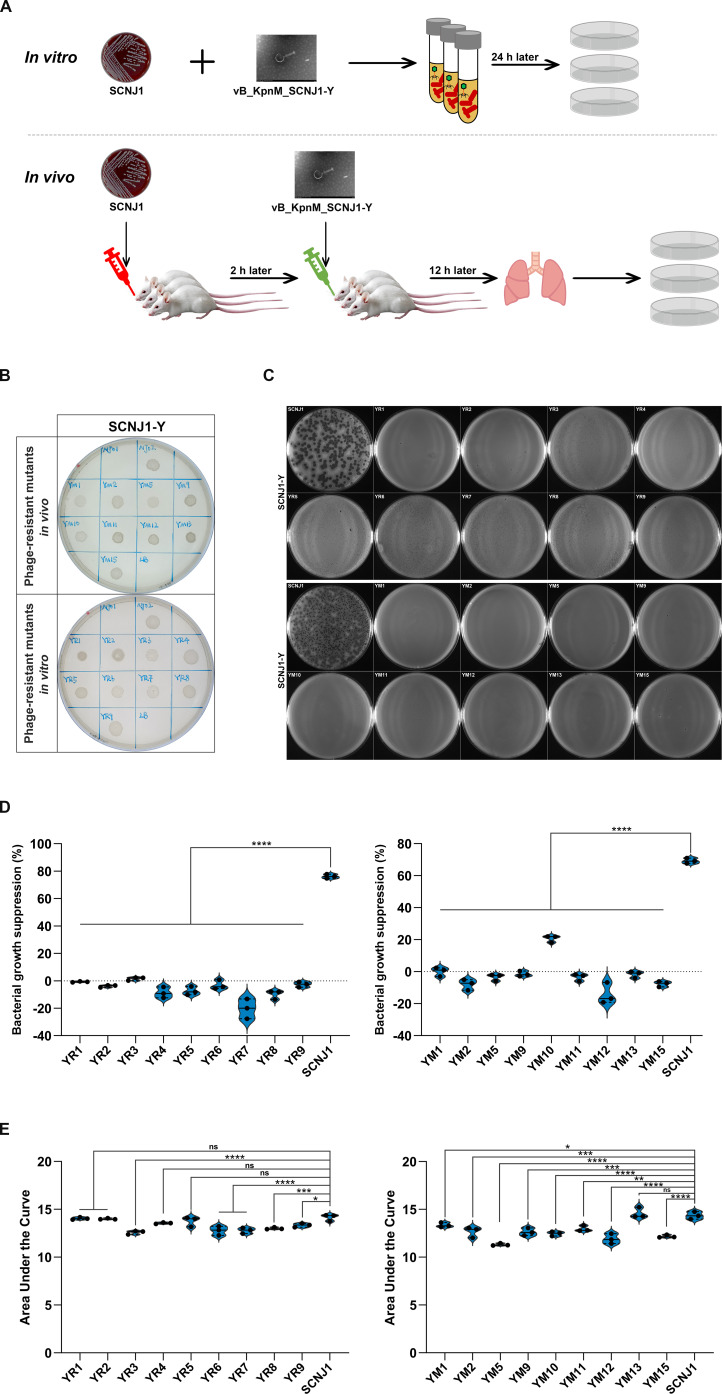
Selection of phage-resistant mutants. (**A**) A schematic showing the phage-host co-evolution experiment. The *K. pneumoniae* strain SCNJ1 was exposed to a single dose of the virulent phage vB_KpnM_SCNJ1-Y (briefly called SCNJ1-Y) *in vitro* within a culture medium or *in vivo* during a phage therapy treatment for murine pneumonia. The images vB_KpnM_SCNJ1-Y are duplicates and originated from our previous study (40). (**B**) Inverted spotting assay. The phage-resistant mutants were grown on the LB agar plate containing 10^9^–10^10^ PFU of phage SCNJ1-Y. *K. pneumoniae* SCNJ1, SCNJ2 (a clinical strain of K47 capsule-serotype *K. pneumoniae*), and LB medium were used as negative control, positive control, and blank control, respectively. (**C**) EOP assay. Phage was inoculated with bacterial culture, and the number of lysis plaques in each strain was measured by the double-layer agar method. Phage formed a circular lysis plaque surrounding the zone of the parental strain SCNJ1 (EOP: 100%), but no plaques were observed on these phage-resistant clones (EOP: 0%). (**D**) Suppression of bacterial growth. The growth curves were converted into the area under the growth curve (AUC) by GraphPad Prism 10.1.2 to evaluate the bacterial growth abilities. The growth suppression of SCNJ1 and phage-resistant clones in the presence of phage (at MOI 0.0001) was expressed as the percentage compared with the AUC of the control (without phage pressure), corresponding to the suppression of the bacterial culture, calculated using the formula: [(AUC of control-AUC of phage treatment)/AUC of control] × 100%. Data shown as mean ± SD (*n* = 3) are from a representative experiment. Data were analyzed by one-way ANOVA with Dunnett’s post hoc test. (**E**) Growth kinetics of SCNJ1 and phage-resistant mutants. Data shown as mean ± SD (*n* = 3) are from a representative experiment. The growth curves and growth inhibition curves at MOI 0.0001 are presented in Fig. S1. Data were analyzed by one-way ANOVA with Dunnett’s post hoc test. *P < 0.05, **P < 0.01, ***P < 0.001, and ****P < 0.0001. ns, not significant.

To explore the underlying molecular mechanisms of phage resistance, we exposed the hypervirulent K54 capsular-type *K. pneumoniae* strain SCNJ1 to a single dose of the virulent phage vB_KpnM_SCNJ1-Y (referred to here as SCNJ1-Y) by coculturing in an LB broth (*in vitro*) or during a phage treatment in a murine pneumonia model (*in vivo*) (Fig. 1A). Phage resistance was determined by inverted spotting plate assays, and evolved resistant clones grew to form bacterial lawns on the plate containing phage SCNJ1-Y (Fig. 1B). Eighteen (nine from *in vitro*, named YR1 to YR9; nine from *in vivo*, named YM1, YM2, YM5, YM9, YM10, YM11, YM12, YM13, and YM15) phage-resistant clones were selected as representatives, and their phage sensitivity was further confirmed by the efficiency of plating (EOP) assay and growth inhibition assays. EOP results indicated that all the evolved clones were completely resistant to phage SCNJ1-Y since their EOP values were all 0% (Fig. 1C). Additionally, phage SCNJ1-Y demonstrated a high level of efficiency in inhibiting the growth of the parental strain SCNJ1. However, it had almost no impact on the growth of the evolved clones (Fig. 1D; Fig. S1).

The effect of phage resistance mutations on the bacterial growth of *K. pneumoniae* strain SCNJ1 was assessed through bacterial growth parameters, namely AUC (Area Under the Curve), in a non-limited LB broth. Most of the resistant clones displayed significantly reduced growth kinetics compared with SCNJ1, indicating their potential fitness burden (Fig. 1E).

### CPS or LPS mutation contributed to phage resistance

For these phage-resistant clones, three different bacterial colony morphology on the LB blood agar plate were observed: (i) mucoid with smooth, big colonies (the parental type, “mb” morphotype, strains YR1, YR2, YM9, YM11, YM12, YM13, and YM15), (ii) non-mucoid with small colonies (“nms” morphotype, strains YR6, YR7, and YM10), and (iii) non-mucoid with medium-size colonies (“nmm” morphotype, the remaining eight strains) (Fig. S2A). String test showed that similar to the parental strain SCNJ1, YR1, YR2, YM9, YM11, YM12, YM13, and YM15 all exhibited a hypermucoviscous phenotype, as evidenced by the viscous strings from their colonies exceeding 5 mm in length (Fig. S2B), indicating a positive string test result. In contrast, the remaining strains all showed negative string test results, which indicates that their mucoviscosity is low. These results imply that the amount or structure of the capsular polysaccharides in these non-mucoid clones may have changed, due to the strong linkage between capsular polysaccharides and hypermucoviscosity (41).

**Fig 2:**
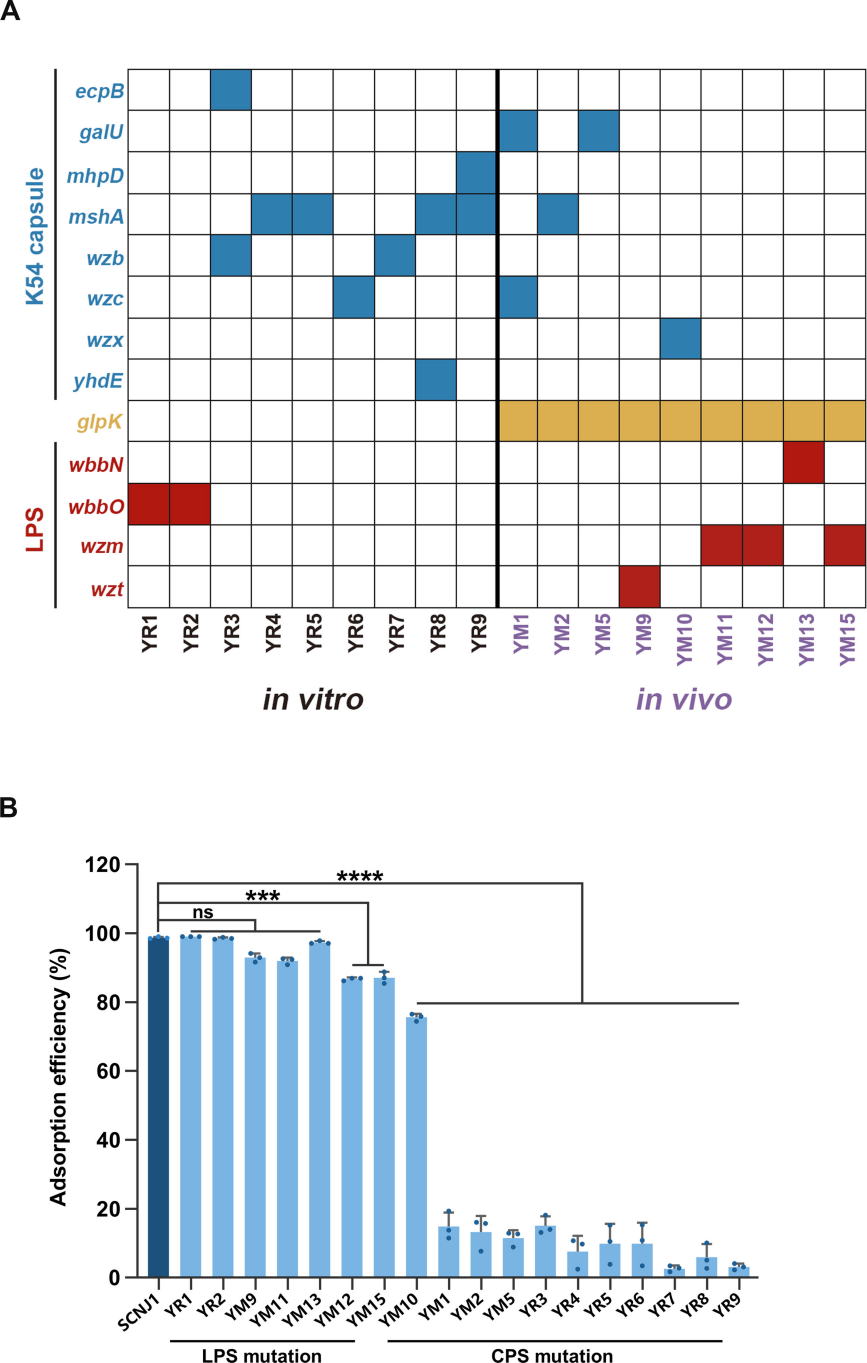
Genetic mutations and phage adsorption tests. (**A**) Distribution of the mutated genes. Genome sequencing of these evolved resistant clones revealed two main mechanisms that drive phage resistance: the mutations related to CPS and those related to LPS. The mutated genes related to CPS and LPS biosynthetic pathways are colored red and blue, respectively. The *glpK* mutation is colored yellow. (**B**) Phage adsorption to resistant clones. The free phage titers in the supernatant were measured after incubation with each evolved clone by the double-layer agar method. The phage adsorption efficiency was calculated as a percentage of phage recovered from the medium-only condition. Strain SCNJ1 served as the positive control. Data are shown as mean ± SD of triplicate samples from at least three independent experiments. Statistical analyses were performed using one-way ANOVA with Dunnett’s post hoc test. ***P < 0.001 and ****P < 0.0001. ns, not significant.

To reveal the genetic basis for phage resistance, we sequenced the genomes of these evolved resistant clones and mapped each of them to the reference genome of the parental strain SCNJ1 (GenBank accession no. CP174529). The high-probability mutations detected in these resistant clones were summarized in Table 1, which shows that each clone carries at least one mutation. All the isolates obtained from *in vivo* samples possessed the *glpK* mutation. It is speculated that this was the result jointly caused by the selective pressures exerted by phages and the mice’s internal environment (25). Two main mechanisms drive phage resistance in these resistant clones. The first was based on the mutations in the CPS biosynthesis pathway, which were detected in 61.11% (11/18) of the phage-resistant clones (Fig. 2A). Consistent with previous studies (13, 14), mutations in the genes of the *cps* operon, including *mshA* (encoding a glycosyltransferase), *wzc* (encoding a tyrosine-protein kinase), *wzb* (encoding a putative capsule polysaccharide export protein), and *wzx* (encoding a teichoic acid export membrane protein), were also frequently detected in our screen. The second was based on the mutations in the LPS (O-antigen) synthesis, and the remaining clones (38.89%, 7/18) each had a single mutation in the O-antigen operon (Fig. 2A). These LPS mutations seem to exhibit a specific association with *in vivo* samples. Specifically, five of the nine *in vivo* samples harbor LPS mutant clones, in contrast to only two of the nine *in vitro* samples (Fig. 2A). The identified mutations are likely to result in the truncation of the LPS or in the impediment of LPS export from the cell. This is because these mutations occur within the LPS O-antigen gene cluster, either in the *wbbN* or *wbbO* gene, which respectively encode glycosyl-transferase and galactosyl-transferase essential for LPS synthesis, or in the *wzm* gene (encoding an O-antigen export system permease protein) or the *wzt* gene (encoding an O-antigen export system ATP-binding protein) that are responsible for the export process of LPS (42). Collectively, these findings suggest that CPS and LPS both act as receptors that facilitate phage binding to, and subsequent infection of, host cells.

**TABLE 1:** Summary of the mutations of phage-resistant clones

Resistant isolate	Gene	Annotation	Putative gene function
YM1	*glpK*	A77A (GCC→GCT^*a*^)	Glycerol kinase
	*wzc*	Δ289 bp	Putative tyrosine protein kinase in *cps* region
	*galU*	Δ1 bp, coding (308/903 nt)	UTP-glucose-1-phosphate uridylyltransferase
YM2	*glpK*	A77A (GCC→GCT)	Glycerol kinase
	*mshA*	Coding (104-118/1161 nt)	Dinositol-3-phosphate glycosyltransferase
YM5	*glpK*	A77A (GCC→GCT)	Glycerol kinase
	*galU*	Δ57 bp, coding (546-602/903 nt)	UTP-glucose-1-phosphate uridylyltransferase
YM9	*wzt*	coding (475/741 nt), IS5 insertion	O-antigen export system ATP-binding protein Wzt
	*glpK*	A77A (GCC→GCT)	Glycerol kinase
YM10	*glpK*	A77A (GCC→GCT)	Glycerol kinase
	*wzx*	(A)_8→7_, coding (628/1224 nt)	Teichoic acid export membrane protein
YM11	*wzm*	coding (276/768 nt), IS5 insertion	O-antigen export system permease protein Wzm
	*glpK*	A77A (GCC→GCT)	Glycerol kinase
YM12	*wzm*	coding (85/768 nt), IS5 insertion	O-antigen export system permease protein Wzm
	*glpK*	A77A (GCC→GCT)	Glycerol kinase
YM13	*glpK*	A77A (GCC→GCT)	Glycerol kinase
	*wbbN*	P94R (CCC→CGC)	Glycosyl transferase
YM15	*wzm*	coding (85/768 nt), IS5 insertion	O-antigen export system permease protein Wzm
	*glpK*	A77A (GCC→GCT)	Glycerol kinase
YR1	*wbbO*	P85Q (CCG→CAG)	Galactosyl transferase
YR2	*wbbO*	P85Q (CCG→CAG)	Galactosyl transferase
YR3	*wzb*	Δ19 bp, coding (580-598/1134 nt)	Putative capsule polysaccharide export protein
	*ecpB*	S47R (AGC→CGC)	putative fimbrial chaperone EcpB
YR4	*mshA*	G298E (GGA→GAA)	Dinositol-3-phosphate glycosyltransferase
YR5	*mshA*	G298E (GGA→GAA)	D-inositol-3-phosphate glycosyltransferase
YR6	*wzc*	Δ1 bp, coding (1669/2151 nt)	Putative tyrosine-protein kinase in cps region
YR7	*wzb*	Δ19 bp, coding (580-598/1134 nt)	hypothetical protein
YR8	*mshA*	coding (498/1161 nt), ISL3 insertion	D inositol 3 phosphate glycosyltransferase
	*yhdE*	G156G (GGT→GGG)	dTTP/UTP pyrophosphatase
YR9	*mshA*	G298E (GGA→GAA)	D-inositol-3-phosphate glycosyltransferase
	*mhpD*	Y163H (TAC→CAC)	2-keto-4-pentenoate hydratase

^*a*^ The bold letters with underlines indicate the mutated bases.

### Capsular polysaccharide was the primary receptor for phage adsorption

The surface-exposed polysaccharide molecule of bacteria plays a pivotal role in phage adsorption (16, 17, 43). For those CPS-related mutants, phage adsorption to them, except for YM10, was significantly reduced to less than 20% when compared with that of the parental SCNJ1 (all, *P*＜0.0001 vs SCNJ1). A likely explanation for the less severe adsorption impairment (75.57% vs 98.70 ± 0.28) to YM10 could be that the mutation in the *wzx* gene of YM10 resulted in a defect in the export of CPS but had little impact on the structural integrity of CPS. In contrast, the adsorption rate of phages to the LPS-related mutants remained at a high level, and the phage adsorption to them was only ~12.0% less than that to SCNJ1 (Fig. 2B). These results suggest that CPS, not LPS, is required for phage adsorption in *K. pneumoniae* strain SCNJ1.

**Fig 3:**
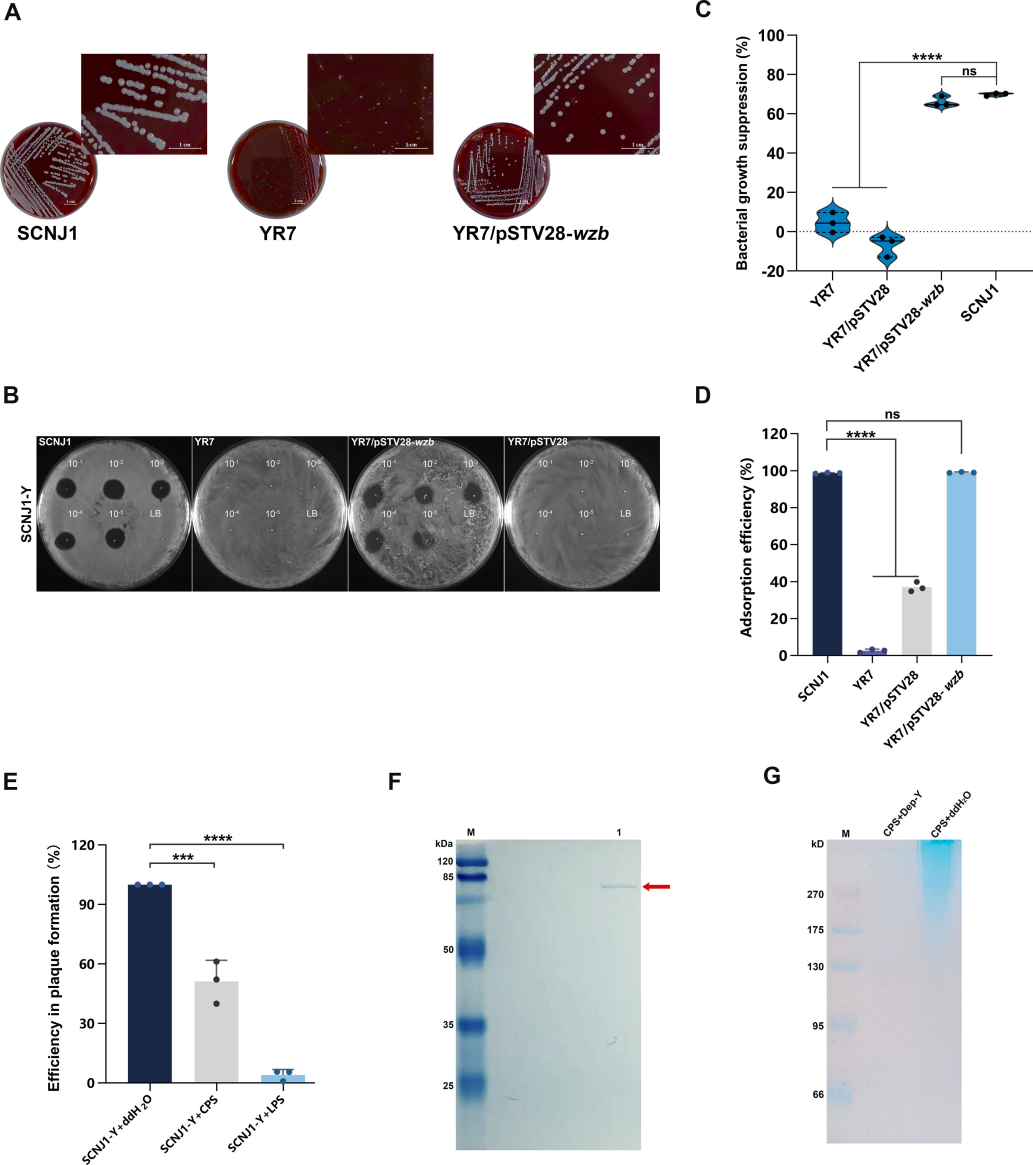
CPS is necessary for phage adsorption and infection. (**A**) Colony appearances of the wild-type SCNJ1, CPS-related mutants YR7 and its complemented strain YR7/pstv28-*wzb* on LB blood agar plates. Boxes indicate areas of increased magnification. (**B**) Representative spot test of SCNJ1, YR7, and YR7/pstv28-*wzb*. Serially diluted phage stock was dropped onto the agar overlaid by the bacterial strain. The presence or absence of a cleared lysis plaque indicated phage sensitivity or resistance. LB broth was used as the negative control. The picture of SCNJ1-Y: SCNJ1 is a duplicate of those in the YR1 and YM13 groups of Fig. 4A, as it is derived from the same experiment as the positive control. (**C**) Growth inhibition assay of YR7 and YR7/pstv28-*wzb*. Data are shown as mean ± SD (*n* = 3) from a representative experiment. Data were analyzed by one-way ANOVA with Dunnett’s post hoc test. ****P < 0.0001. ns, not significant. (**D**) Phage adsorption test. The adsorption efficiency of phage to the bacterial cell was calculated as a percentage of phage recovered from the medium-only condition. Strain SCNJ1 served as the positive control. Data are shown as mean ± SD of triplicate samples from three independent biological replicates. Statistical analyses were performed using one-way ANOVA with Dunnett’s post hoc test. ****P < 0.0001. ns, not significant. (**E**) Phage infection inhibition assay. Using the *K. pneumoniae* strain SCNJ1 as the host bacteria, the phage titers were determined by the double-layer agar method after the phages were incubated with purified CPS or LPS. Data are shown as mean ± SD of triplicate samples from three independent biological replicates. Statistical analyses were performed using one-way ANOVA with Dunnett’s post hoc test. ***P < 0.001, and ****P < 0.0001. (**F**) Protein expression of Dep-Y. Recombinant protein Dep-Y was separated by 12% SDS-PAGE along with Coomassie blue staining. Lane M, protein marker; Lane 1, purified Dep-Y. (**G**) Capsule degradation assay and alcian blue staining. CPS of strain SCNJ1 was treated with protein Dep-Y or an equal volume of ddH_2_O (control). After separation by 8% SDS-PAGE, the CPS phenotypes were visualized by alcian blue staining.

To verify that the mutations in the CPS gene cause phage resistance and phage SCNJ1-Y utilizes CPS as the preliminary adsorption receptor, YR7 was selected as the representative CPS-related mutant. We cloned the wild-type version of the *wzb* gene from strain SCNJ1 into YR7, assigned YR7/pSTV28-*wzb*. As expected, the colonies of YR7/pSTV28-*wzb* restored to the “mb” morphotype (Fig. 3A). Spot test, EOP assay, and growth inhibition assays revealed that YR7/pSTV28-*wzb* successfully restored its susceptibility to phage SCNJ1-Y (Fig. 3B and C; Table S1; Fig. S3 and S4). Adsorption assay showed that in contrast to the mere 2.61% adsorption rate of phage SCNJ1-Y to strain YR7, the adsorption rate of SCNJ1-Y to YR7/pSTV28-*wzb* reached 99.01%, comparable with the 98.7% of the parental strain SCNJ1 (Fig. 3D), suggesting that the integrity of CPS was important for phage adsorption to bacteria.

To further verify the important role of CPS during phage infection, purified CPS of SCNJ1 was employed for the infection inhibition experiment. We found that incubation of phage SCNJ1-Y with CPS before infection of strain SCNJ1 significantly reduced the number of plaque-forming units (infection efficiency, 51.17% vs 100%, *P* = 0.0001) (Fig. 3E), indicating the added CPS competitively bound to the receptor-binding protein of CPS on the phage.

It has been known that phages encode a depolymerase that can recognize and bind to bacterial CPS to degrade it (37). Here, we recombinantly expressed the putative depolymerase Dep-Y of phage SCNJ1-Y via a pET-22b expression system with an N-terminal hexahistidine tag. SDS-PAGE showed the recombinant protein Dep-Y to have a size of ~74 kD (Fig. 3F). SDS-PAGE and Alcian blue staining of the CPS that had been treated with Dep-Y demonstrated the enzymatic activity of Dep-Y against the K54 CPS polymer (Fig. 3G). These results collectively suggest that CPS serves as a receptor for phage adsorption, and depolymerase Dep-Y of the phage can recognize and degrade CPS, which is essential for the first committed step of phage infection of its host.

### LPS was the secondary receptor for phage genome DNA injection

**Fig 4:**
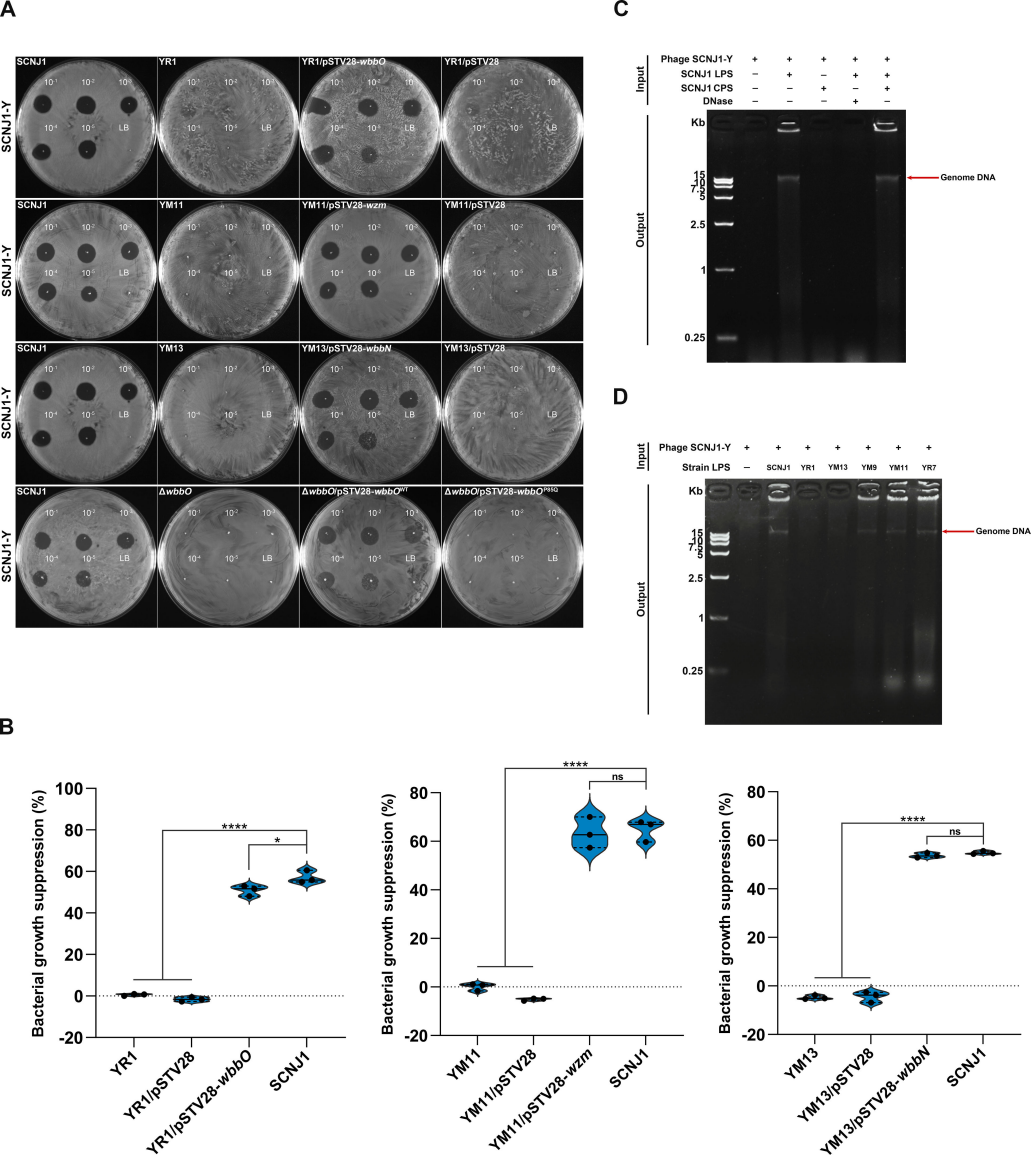
LPS is essential for phage DNA ejection. (**A**) Representative spot test. Serially diluted phage stock was dropped onto the agar overlaid by the bacterial strain. LB broth was used as the negative control. The presence or absence of cleared lysis plaque indicated phage sensitivity or resistance. The pictures of SCNJ1-Y: SCNJ1 in the YR1 and YM13 groups are duplicates, as they are derived from the same experiment as the positive control. (**B**) Growth inhibition assays of LPS-related mutants and their complementation strains. The growth suppression of bacterial strains in the presence of phage (at MOI 0.0001) was calculated using the formula: ([AUC of control − AUC of phage treatment]/AUC of control) × 100%. Data are shown as mean ± SD (*n* = 3) from a representative experiment. Statistical analyses were performed using one-way ANOVA with Dunnett’s post hoc test. *P < 0.05 and ****P < 0.0001. ns, not significant. (**C**) LPS-mediated DNA release from phage SCNJ1-Y observed *in vitro*. Phage particles were incubated with LPS of the strain SCNJ1, and the released product was detected on a 1.2% agarose gel electrophoresis along with ethidium bromide staining. Upon incubation with LPS, the genomic DNA of phage particles was released, and the ejected DNA was sensitive to DNase digestion. CPS alone could not trigger the DNA release. (**D**) The release of phage DNA required intact LPS. The defective LPS in YR1 and YM13 failed to trigger the DNA release.

To confirm that mutations in the LPS gene cause phage resistance, we cloned wild-type *wbbO*, *wbbN*, and *wzm* into YR1, YM13, and YM11, respectively. Spot test, EOP assay, and growth inhibition assays showed that the susceptibility to phage SCNJ1-Y was successfully restored in the trans-complemented strains YR1/pSTV28-*wbbO*, YM13/pSTV28-*wbbN*, and YM11/pSTV28-*wzm* (Fig. 4A and B; Table S1; Fig. S5 and S6). To further verify the necessary role of the LPS gene in bacterial phage resistance, the *wbbO* gene deletion was conducted in strain SCNJ1 (named Δ*wbbO*). We found that the deletion of *wbbO* abolished plaque formation, whereas the complementation of the wild-type *wbbO* (*wbbO*^WT^), but not the mutated *wbbO^P85Q^*, restored the phage susceptibility to phage SCNJ1-Y (Fig. 4A; Table S1; Fig. S5). These results demonstrate that the mutations in the LPS gene are a decisive factor in contributing to phage resistance and that the integrity of LPS is important for phage infection.

To further verify that LPS is essential for phage infection, an infection inhibition experiment was conducted using purified LPS of SCNJ1. We found that the pre-incubation of phage SCNJ1-Y with LPS significantly reduced its efficiency in plaque formation (4.01% vs 100%, *P* < 0.0001) (Fig. 3E), indicating that LPS is a binding receptor during phage infection.

It has been established that the interaction of phage with host LPS is responsible for initial phage adsorption in many bacterial species, such as *E. coli* (20), *P. aeruginosa* (44), and *Salmonella Typhimurium* (45). However, we found that the phage adsorption efficiency of LPS mutants (such as YM9 and YR1) was comparable with that of the parental strain SCNJ1 (Fig. 2B), revealing that LPS has little effect on the phage adsorption process in this strain. Previous studies showed that LPS can serve as a receptor that triggers phage DNA ejection into the cells to initiate infection (46–48). This prompted us to elucidate the essential role of LPS of SCNJ1 in phage DNA ejection. We performed DNA release assays *in vitro* and analyzed phage preparations before and after their incubation with purified LPS alone, a mixture of LPS and CPS, or CPS alone on ethidium bromide-stained agarose gels. After incubation of phage SCNJ1-Y with LPS, but not CPS, the free phage DNA was released, resulting in a band at a position ~15 kb marker (Fig. 4C). This result shows that LPS alone, independent of CPS, can trigger DNA ejection. The DNA band disappeared after the treatment with DNase I, confirming the DNA accessibility.

To demonstrate the necessity of intact LPS in triggering phage DNA ejection, purified LPS of LPS-related mutants YR1(*wbbO*^mut^), YM11(*wzm*^mut^), YM13(*wbbN*^mut^), and CPS-related mutant YR7(*wzb*^mut^) were prepared. DNA release assays demonstrated that the LPS of YR1 and YM13 couldn't start phage DNA ejection, whereas those of YM11 and YR7 did (Fig. 4D), indicating that intact LPS is essential for phage DNA ejection. The LPS-related mutant YM11 retained the ability to facilitate the release of phage DNA, which is likely due to the fact that its mutant gene *wzm* is associated with the export of LPS and has no impact on the LPS biosynthesis. These results collectively suggest that LPS is most likely to act as a “secondary receptor” for phage, mediating the phage infection process by triggering the release of the phage genome.

### LPS was recognized by a novel tail fiber protein

**Fig 5:**
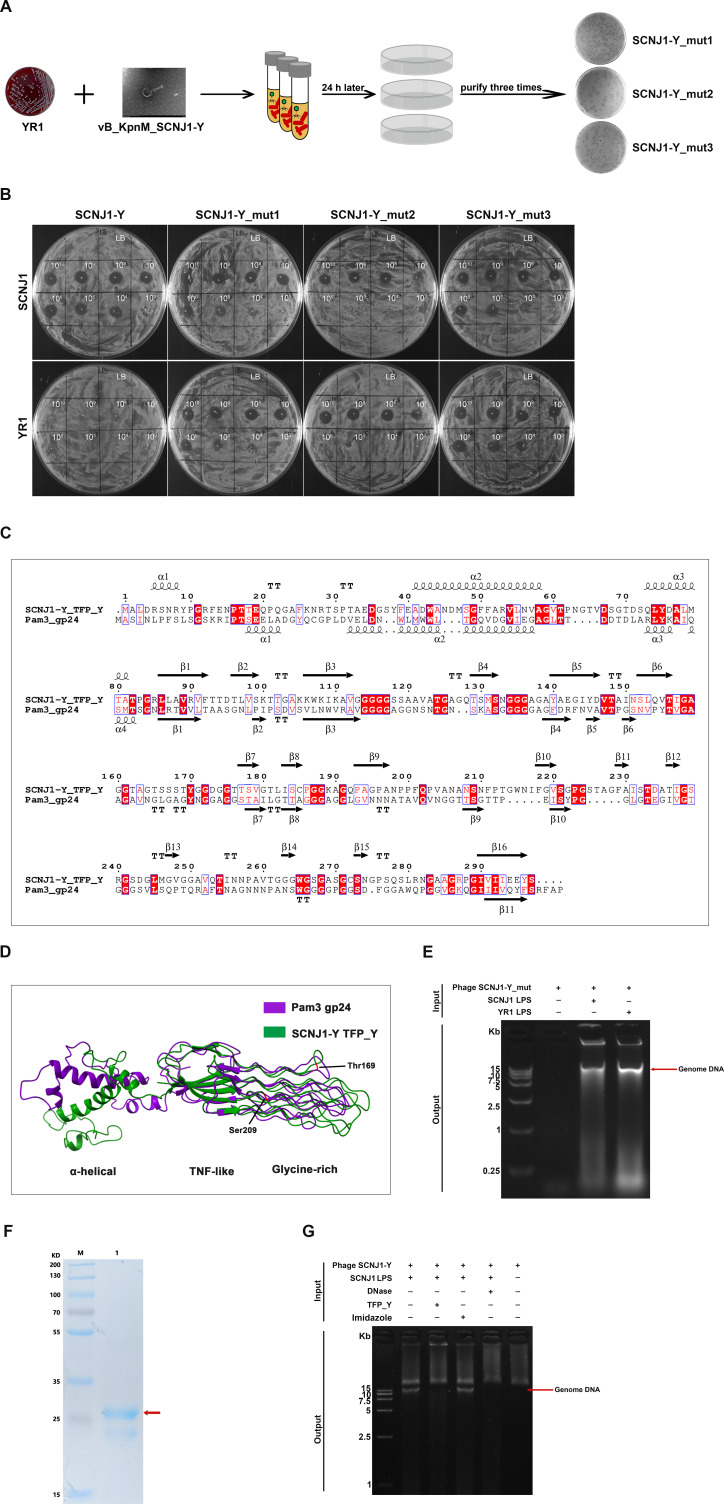
LPS is recognized by a novel tail fiber protein. (**A**) A schematic showing the isolation of mutant phages. The phage SCNJ1-Y and YR1 (an LPS-related mutant) were cocultured in liquid culture, and the evolved mutant phages that can infect YR1 were isolated and purified by the double-layer agar method. (**B**) Representative spot test. Serially diluted phage stock of parental SCNJ1-Y or mutant phage (SCNJ1-Y_mut1, SCNJ1-Y_mut2, and SCNJ1-Y_mut3) was dropped onto the agar overlaid by strain SCNJ1 or YR1. The presence or absence of cleared lysis plaque indicated phage sensitivity or resistance. (**C**) Alignment of amino acid sequences of TFP_Y and gp24 of phage Pam3. Secondary structures of TFP_Y predicted by SWISS-MODEL are represented with coils denoting α-helices and arrows denoting β-strands. Identical residues across sequences are marked against a red background, and homologous residues are highlighted in red. (**D**) Structural comparisons of TFP_Y and the tail fiber protein gp24 of phage Pam3 with homologs. The structures are shown in a cartoon and colored green for TFP_Y and purple for gp24. The amino acid loci where mutations occur in TFP_Y, Thr169 and Ser209, are marked in red. (**E**) LPS of strain YR1 triggered the DNA release of mutant phage. LPS of SCNJ1 and ddH_2_O served as the control. (**F**) Protein expression of TFP_Y. Recombinant protein TFP_Y was separated by 12% SDS-PAGE and stained with Coomassie blue. Lane M, protein marker; Lane 1, purified TFP_Y. (**G**) TFP_Y inhibited LPS-triggered phage DNA ejection. Phage particles were incubated with LPS of SCNJ1 and phage protein TFP_Y (suspended in 50 mM imidazole solution), and the released product was detected on a 1.2% agarose gel electrophoresis along with ethidium bromide staining. In the presence of TFP_Y, LPS failed to initiate the release of the phage genome. An equal volume of 50 mM imidazole solution was used as a control.

Many tailed phages use RBPs at the distal end of their tail to specifically recognize and interact with receptors on the bacterial cell surface, such as LPS (33). Phages can evolve to overcome bacteria that have mutated phage receptors by mutating their RBPs. Here, we performed coevolution experiments using the LPS-related phage-resistant mutant YR1 and the phage SCNJ1-Y (Fig. 5A). After their co-cultivation, three distinctive plaques formed on the bacterial lawn of strain YR1 were picked. Three pure regained-infectivity phage mutants (named SCNJ1-Y_mut1, SCNJ1-Y_mut2, and SCNJ1-Y_mut3) were obtained by propagating them on strain YR1 successively three times (Fig. 5A). Their infectivity to YR1 was further confirmed by spot test (Fig. 5B).

Whole-genome sequencing of these phage mutants revealed non-synonymous SNPs at two identical loci of the same gene *tfp_Y*, which encodes a putative tail fiber protein, named TFP_Y (Table S2; Fig. S7). The mutated TFP_Y protein (T169I, S209G) restored the infectivity of phage SCNJ1-Y to the LPS-mutant strain, implying that protein TFP_Y may serve as the RBP of LPS. The *tfp_Y* gene encodes 297 amino acids with a predicted molecular weight of 29.3 kDa. BLASTp analysis against the NCBI database revealed the sequence conservation of TFP_Y among *Klebsiella* phages, indicating that TFP_Y had a conserved function. However, it showed no sequence similarity to the previously reported tail fiber or tail spike proteins responsible for triggering DNA ejection, including *gp21* of *Bacillus subtilis* phage SPP1 (49), *gp17* of *E. coli* phage T7 (48), and *gp9* of *Salmonella* phage P22 (46). Swiss-Model homology showed that TFP_Y had the highest similarity (GMQE 0.53, 80% coverage, 32% identity) to the tail fiber gp24 of a newly reported *Myoviridae* cyanophage Pam3 (50). Amino acid sequence alignment of TFP_Y and gp24 of Pam3 revealed several conserved residues (Fig. 5C). Structural similarity search using DALI revealed that the structure of TFP_Y most resembles the tail fiber gp24 (PDB ID: 7yfw) of Pam3 (50, 51), with a root-mean-square deviation (RMSD) of 4.4 Å over 280 Cα atoms (Fig. 5D). They both contain three distinct domains, with the TNF-like domain and glycine-rich domain sharing a similar architectural structure that is presumed to recognize various host receptors. However, there is a significant discrepancy in the N-terminal α-helical domain, which plays a critical role in docking to the phage baseplate (27). In this work, the amino acid loci where mutations occur in TFP_Y, Thr169 and Ser209, are both located in the glycine-rich domain, which could explain the host adaptation of the mutant TFP_Y. The mutations at Thr169 and Ser209 of TFP_Y restored bacterial sensitivity to the phage, indicating that these residues are likely to be involved in LPS binding or catalysis. The critical role of these two loci in phage-host interaction warrants further experimental validation.

To determine whether LPS triggers the release of phage DNA by interacting with the TFP_Y protein of phage SCNJ1-Y, DNA ejection assays were performed. Although LPS of YR1 was unable to trigger the DNA ejection of the parental phage SCNJ1-Y (Fig. 4D), it effectively initiated the triggering event of the mutant phage SCNJ1-Y_mut2 (Fig. 5E). This result suggests that the mutated TFP_Y protein in phage SCNJ1-Y_mut2 has adapted to the mutated LPS of strain YR1 and that the TFP_Y protein is most likely to recognize and bind to LPS during phage infection. Furthermore, we found that the trigger of DNA ejection of phage SCNJ1-Y_mut2 could also be initiated by the LPS of wild-type strain SCNJ1, indicating that the mutations in the TFP_Y protein expanded the host range of phage SCNJ1-Y.

To further confirm the interaction of phage TFP_Y protein with host LPS, the putative phage tail fiber gene *tfp_Y* was cloned and expressed via a pET-22b expression system (Fig. 5F). The recombinant protein TFP_Y was used for the DNA ejection inhibition assay. We found that LPS pretreated with TFP_Y protein was no longer able to trigger phage DNA ejection, indicating that TFP_Y has prevented LPS-triggered phage DNA ejection (Fig. 5G). Taken together, these results support the notion that protein TFP_Y is the RBP that interacts with host LPS and is necessary to initiate DNA release of phage SCNJ1-Y.

### The fitness cost of phage-resistant clones

**Fig 6:**
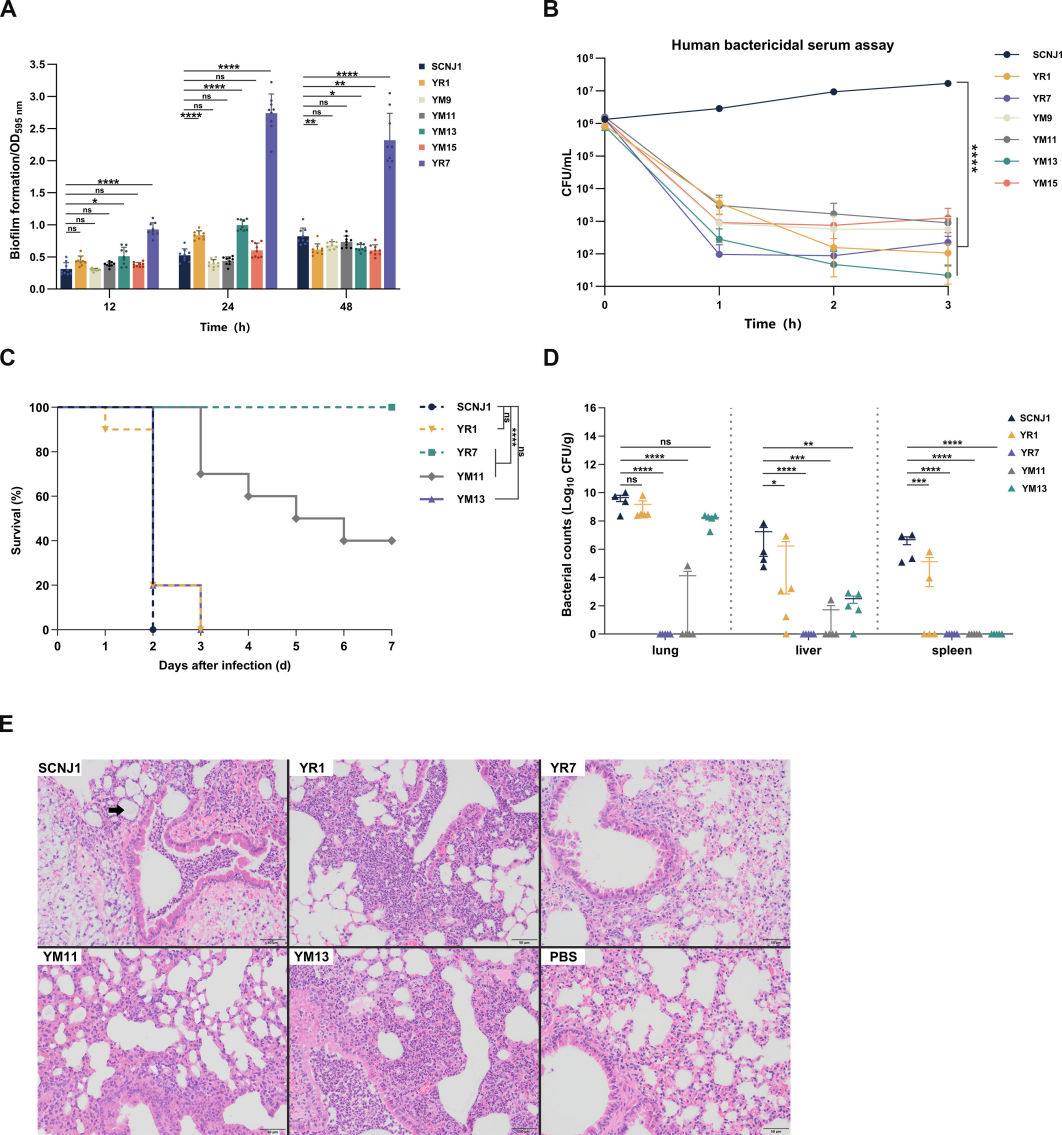
Fitness costs of representative phage-resistant mutants. (**A**) Quantification of biofilm formation. The biofilms were cultured in a 96-well plate and stained with crystal violet staining at 12 h, 24 h, and 48 h. Data are shown as mean ± SD from three independent biological replicates in triplicate. Statistical analyses were performed using two-way ANOVA. (**B**) The serum killing assays. Bacteria and human serum from healthy volunteers were mixed at a 1:3 vol/vol ratio. At the time points of 0, 1, 2, and 3 h, the viable bacteria were counted. Data are shown as mean ± SD from three independent biological replicates in triplicate. Statistical analyses were performed using two-way ANOVA. (**C**) Survival curves. Mice (*n* = 10 for each strain) were intraperitoneally inoculated with 2 × 10^7^ CFU of either the wild-type SCNJ1 or phage-resistant mutant and were monitored for 7 days. Statistical analyses were performed using the log-rank (Mantel-Cox) test. (**D**) The organ burden assays. Mice (*n* = 5 for each strain) were intraperitoneally inoculated with 1 × 10^6^ CFU of either the wild-type SCNJ1 or phage-resistant mutant; 48 h after infection, the bacterial loads in the liver, lungs, and spleen of mice were counted. Each symbol represents one animal, and error bars denote standard deviations of the mean. The organ burden was standardized with the number of bacteria per 1 g wet organ weight. Statistical analyses were performed using one-way ANOVA with Dunnett’s post hoc test. *P < 0.05, **P < 0.01, ***P < 0.001, and ****P < 0.0001. ns, not significant. (**E**) Histopathology analysis. The images depict HE-strained lung tissues of mice at 48 h after infection with 1 × 10^6^ CFU of either SCNJ1, phage-resistant mutant, or PBS (negative control). These images represent three sections from three mice, all showing similar tissue pathology. The bacteria in the bronchial lumen are pointed out with a black arrow.

To evaluate whether phage-resistant mutations engender a fitness cost, five LPS-related mutants (YR1, YM9, YM11, YM13, and YM15) and one CPS-related mutant (YR7) were selected as representative strains for further analysis. First, we quantified the biofilm formation of the wild-type strain and phage-resistant mutants at three time points (12 h, 24 h, and 48 h). YR7, which carries a single mutation in the CPS gene, produced a large amount of biofilm, significantly higher than the wild-type SCNJ1 (*P* < 0.0001 vs SCNJ1, Fig. 6A). This result suggests that the loss of CPS imposed by phage selection promoted bacterial biofilm formation. In contrast, the biofilm formed by LPS-related mutants is at a low level. Compared with the wild-type strain, the amount of biofilm in these mutants either increased slightly or remained unchanged at each time point, and there were only rare cases where it decreased (Fig. 6A). This result indicates that these LPS mutations had little impact on the biofilm-forming ability of bacteria.

Next, we tested the bacterial survival ability of phage-resistant mutants in human serum. The wild-type SCNJ1 survived the exposure to 75% serum over a 3 h incubation period at 37°C, whereas all the phage-resistant mutants exhibited significantly decreased growth ability in serum (all, *P* < 0.0001 vs SCNJ1, Fig. 6B). These results indicate that the mutations selected by phage resistance make the bacteria more vulnerable to the complement-mediated serum killing and that LPS and CPS, both being envelope components, are crucial for bacteria to survive in serum.

To assess the virulence of phage-resistant mutants *in vivo*, we intranasally inoculated BALB/c mice (*n* = 10 for each strain) with 2 × 10^7^ CFU of mutants or wild-type SCNJ1 (as a control), and the survival rate of the mice was continuously monitored for 7 days. After 48 h of infection, the survival rate of mice infected with SCNJ1 dropped to 0, whereas that of mice infected with YR1 or YM13 was 20%. In contrast, mice infected with strain YR7 or YM11 remained alive (Fig. 6C). Besides, mice infected with strain YR1 or YM13 all died by day 3, whereas those infected with strain YR7 survived over 7 days (YR1 vs SCNJ1, *P* = 0.5151; YM13 vs SCNJ1, *P* = 0.1462; YR7 vs SCNJ1, *P* < 0.0001); 30% of YM11-infected mice died on the third day, and the mortality increased to 40% by day 4, reached 50% by day 5, and rose to 60% by day 6 after infection. On the seventh day, no more mice died (YM11 vs SCNJ1, *P* < 0.0001, Fig. 6C). These findings demonstrate that the mutation in the gene encoding CPS synthesis (strain YR7) results in substantial attenuation in the virulence of hypervirulent *K. pneumoniae*. The mutation in the gene related to LPS export (strain YM11) caused partial attenuation. In contrast, strains YR1 and YM13 have altered or defective LPS, but their virulence is not significantly impaired.

To evaluate the *in vivo* colonization and dissemination ability of phage-resistant mutants, we intranasally inoculated BALB/c mice (*n* = 5 for each strain) with 1 × 10^6^ CFU of mutants or SCNJ1 (as a control). After 48 h post-infection, organs (lung, liver, and spleen) were dissected, and bacterial titers were quantified. As shown in Fig. 6D, in the lung, YR1 and YM13 produced bacterial loads at levels comparable with those of the wild-type SCNJ1 (YR1 vs SCNJ1, *P* = 0.7493; YM13 vs SCNJ1, *P* = 0.2275). In contrast, the lung bacterial loads in YR7- and YM11-infected mice were significantly lower than those of mice infected with SCNJ1 (both *P* < 0.0001 vs SCNJ1), with the mean level of CFU (±SD) in mice lungs 10-log lower for YR7 (0.30 ± 0) and YM11 (1.21 ± 2.03) compared with WT strain (9.37 ± 0.72), indicating that YR7 and YM11 had significantly reduced ability to colonize in mice. Furthermore, our findings revealed that the *in vivo* dissemination ability of those phage-resistant mutants had significantly declined, as they yielded substantially lower bacterial titers in the liver and spleen of mice than SCNJ1 did (liver, *P* < 0.05 vs SCNJ1; spleen, *P* < 0.001 vs SCNJ1).

Histopathological analysis was performed on mouse lungs prepared 48 h after infection. Similar to those infected with SCNJ1, lungs infected with YR1 or YM13 demonstrated a large number of neutrophils and bacteria in the bronchial lumen. In the lung tissue around the bronchi, some alveolar septa are thickened, and capillaries are dilated and congested (Fig. 6E). In YM11-infected lungs, there is an infiltration of inflammatory cells mainly consisting of lymphocytes around the bronchi. The alveolar septa are thickened, and the alveolar capillaries are dilated and congested. In contrast, the histologic findings of YR7-infected lungs were nearly normal, and the alveolar septa were only slightly thickened (Fig. 6E).

## DISCUSSION

hv*Kp* is a virulent bacterial pathogen that is capable of causing severe organ and life-threatening diseases, including healthy individuals of any age (4). Phage treatment proves highly promising for patients infected with hv*Kp*. One obvious limitation of phage therapy is the evolution of phage resistance in bacteria (11). Laboratory characterization of phage-resistant mutants could help optimize clinical phage selection and cocktail design. In this study, phage SCNJ1-Y was used to exert selective pressure on the hv*Kp* strain SCNJ1 under both *in vitro* and *in vivo* conditions. Sequencing and comparative genomics of phage-resistant clones revealed mutations in two mechanisms, CPS and LPS biosynthesis pathways, implying that both the CPS and LPS are necessary for efficient phage infection.

Our findings reveal that a mutation in the genes of the *cps* operon largely contributed to the archiving of phage resistance. These CPS-related mutants exhibited non-mucoid with smaller colonies compared with the parental SCNJ1, indicating loss of or reduction in the content of CPS. Another mechanism responsible for phage resistance is the mutation of genes within the LPS O-antigen gene operon, which includes those genes encoding for the synthesis and export of O-antigen. In contrast to the pronounced changes in the colony morphology of the CPS mutants, the colony morphology of the LPS mutant strain shows no significant differences compared with that of the parental strain. Interestingly, we found that the LPS mutation imposed by phage selection of SCNJ1-Y seems to exhibit more association with the *in vivo* samples. This might be attributed to the fact that CPS is crucial for bacteria to evade the body’s immune system. Thus, mutants with compromised CPS are more likely to be cleared by the immune response, increasing the probability of isolating LPS-mutant strains from *in vivo* samples.

For the phage-resistant bacteria, the phage could not kill them directly. However, the development of phage resistance is often accompanied by reduced fitness, which directs bacterial populations toward a more favorable therapeutic direction, rendering the concept of “phage steering” (52). Consistent with previous reports, our CPS-deficient mutants exhibited high fitness costs, including compromised serum survival ability, attenuated virulence, and reduced colonization in mice. Since CPS is essential in shielding bacteria from the host’s immune responses, and hv*Kp* is typically characterized by the overproduction of CPS (53), the evolutionary trade-off between phage resistance and deficient CPS synthesis is beneficial for antibacterial therapies against hv*Kp* infections. However, the virulence assay demonstrated that mutants with defective LPS retained virulence levels comparable with the wild-type strain, and the virulence of those strains that were unable to export LPS was only partially attenuated. For “phage steering,” the resulting virulence phenotype caused by LPS-related mutation has no positive effect on treatment. Fortunately, the mutation of LPS leads to a significant decrease in the bacteria’s resistance to serum killing and a reduction in their ability to spread within mice. In this case, we may potentially improve treatment outcomes by locally applying phage-antibiotic synergy, thereby reducing the side effects of systemic administration of antibiotics.

The phage infection process starts with the adsorption step, during which the phage binds to receptors on the bacterial cell surface (16). In *E. coli*, K15 capsule mutations confer phage resistance by overproducing capsule synthesis to physically mask the phage receptor (20). In contrast to this finding, our CPS mutants exhibited reduced capsule synthesis, and the loss or defect of CPS greatly inhibited phage adsorption capacity, indicating that in *K. pneumoniae*, CPS is the specific adsorption receptor for phages (12, 14, 54), not just a protective shelter for phage-binding domains. CPS can be cleaved by phage-encoded depolymerase, leading to exposure of the LPS (55). We found that the added LPS could inhibit phage infection, revealing that LPS is also a phage-binding receptor in *K. pneumoniae*. So far, the roles of LPS and CPS when they act as receptors simultaneously during the phage infection process have not been reported yet in *K. pneumoniae*. As LPS had little effect on phage adsorption, we shifted our focus to exploring the roles of LPS in mediating phage DNA injection. The results showed that LPS of strain SCNJ1 alone could trigger the release of the phage genome, indicating that no other receptors are involved in this process. These results collectively suggest that CPS is the primary receptor responsible for initial phage adsorption, and LPS is the secondary receptor essential for triggering phage genome injection in *K. pneumoniae* SCNJ1. Therefore, LPS and CPS mutants employ distinct mechanisms to confer phage resistance. Mutations in CPS may prevent phage from entering the cell by altering or losing surface phage receptors, whereas mutations in LPS will impede the successful injection of the phage genome.

Tailed phages recognize and bind to host cell surface receptors with the assistance of RBPs, such as tail fibers and tail spike proteins, enabling the phage particle to initiate the process of irreversible attachment and the subsequent genome injection (46, 56). Our findings showed that the K54 capsular of strain SCNJ1 can be recognized and cleaved by phage-encoded tail spike protein, the depolymerase Dep_Y, indicating that Dep_Y is likely the RBP interacting with CPS of strain SCNJ1. To identify the RBP of LPS, we performed the coevolution experiments using the LPS mutant as the host. As the LPS mutation in the host strain can be overcome by mutations in phage RBPs during the evolution process, we analyzed the genome sequences of the evolved phage and identified mutations in the putative tail fiber gene *tfp_Y*. The homology modeling showed that TFP_Y had the highest similarity to the tail-fiber protein gp24 in the related phage Pam3. A recent study by Thung *et al.* found that mutations in the gp24-homologous tail-fiber protein (MMNM_27) in phage MMNM(Ala_134_) (another Pam3-related phage) rendered the phage binding its LPS receptor of its initially semi-permissive *Klebsiella* host (21). In this study, we found that the evolved phage with mutated *tfp_Y* was able to respond to the signals of the mutated LPS and successfully initiated the delivery process of the phage genome. Furthermore, the TFP_Y protein can inhibit LPS-triggered phage DNA ejection. These findings collectively demonstrate that the gp24-homologous tail-fiber protein is an RBP that interacts with LPS during phage infection of *Klebsiella*.

**Fig 7:**
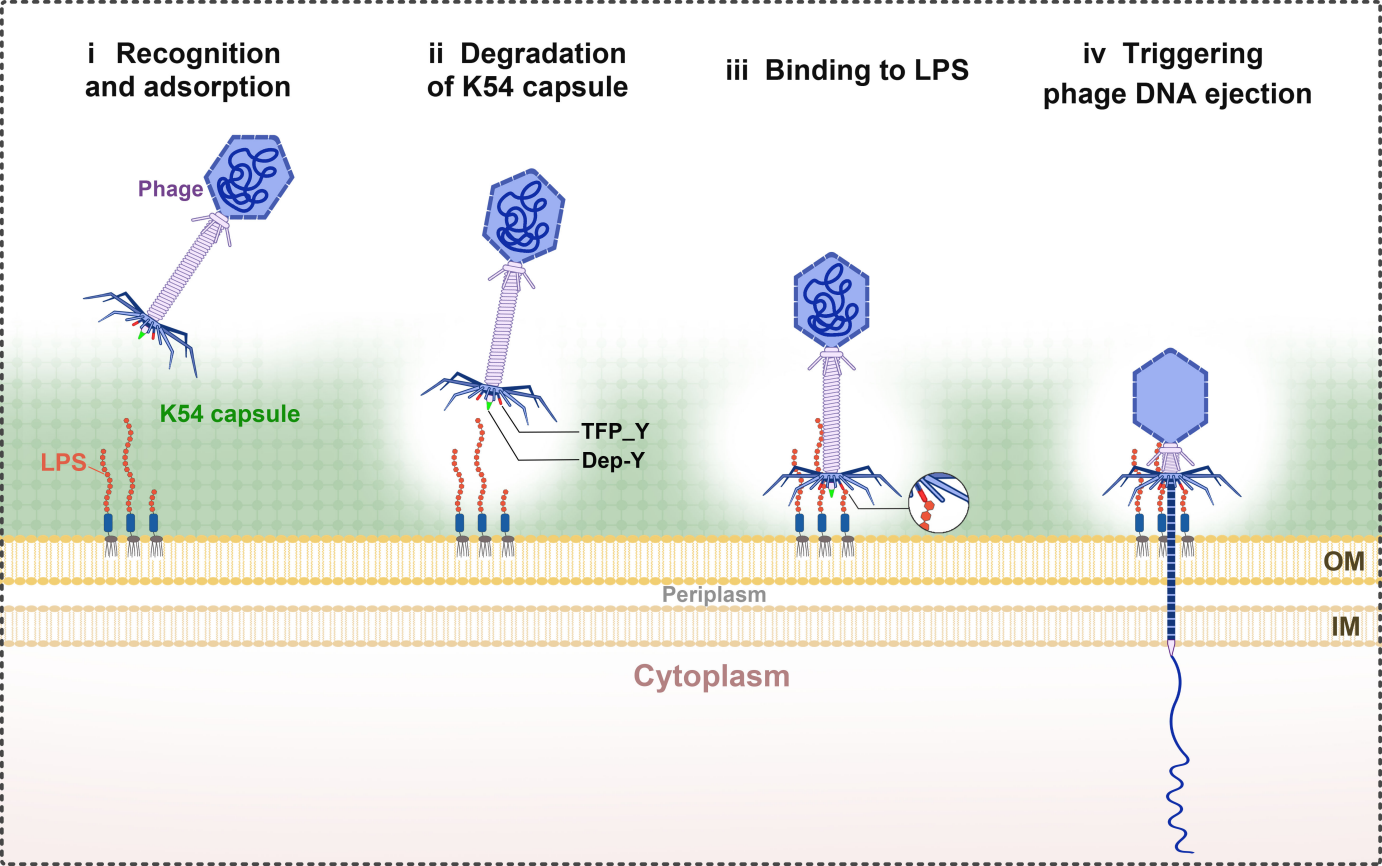
Model of phage SCNJ1-Y targeting of K54-type hv*Kp* strain SCNJ1**.** First, phage recognizes and attaches to the bacterial cell surface. Second, phage-encoded depolymerase Dep_Y specifically degrades the protective capsule, allowing for access to LPS on the bacterial cell wall. Third, the phage binds to LPS. Fourth, the interaction of phage tail fiber protein TFP_Y with LPS triggers phage genome release into the host. OM, outer membrane; IM, inner membrane.

Notably, we found that those known RBPs that interact with LPS and mediate the phage genome delivery, such as *gp17* in *E. coli* phage T7 (48) and *gp9* in *Salmonella* phage P22 (46), have a molecular weight of approximately over 60 kD. However, the molecular weight of protein TFP_Y is only around 29 kD, which is significantly smaller than that of the known RBPs. We speculate that TFP_Y might represent a novel, small phage RBP that interacts with the host LPS to initiate the release of the phage genome. Based on these findings, we proposed a model for the infection process of phage SCNJ1-Y on K54 hv*Kp* strain SCNJ1 (Fig. 7). First, the phage recognizes and attaches to the bacterial cell surface. Second, the capsule is partially degraded by the phage depolymerase Dep_Y. Third, the active site of LPS is exposed, which is recognized by phage tail fiber protein TFP_Y. Fourth, the binding of TFP_Y with LPS facilitates phage DNA ejection.

We acknowledge the limitations of this study. First, more diverse *in vitro* phage-bacteria co-culturing conditions or animal models, and a larger number of independent bacterial cultures combined with pool-sequencing are expected to better elucidate the phage resistance mechanisms. Second, we identified a novel tail fiber protein TFP_Y that interacts with the LPS of *K. pneumoniae* and triggers the ejection of the phage genome. However, the mode of action between TFP_Y and LPS remains to be elucidated. Will the TFP_Y enzymatically hydrolyze the LPS or simply bind to it? The exact underlying mechanisms warrant further exploration.

In conclusion, our findings demonstrate that the *myovirus* phage SCNJ1-Y infects K54-type hv*Kp* strain SCNJ1 by successively recognizing CPS and LPS. This host recognition mechanism explains the phage resistance that arises via mutations in the CPS or LPS. A deeper understanding of phage infection mechanisms would help maximize the efficacy of clinical phage therapy.

## MATERIALS AND METHODS

### Bacterial strain, phage, growth conditions, and media

Strains, phages, and plasmids used in this study are listed in Table 2. *K. pneumoniae* strain SCNJ1, an ST29-K54 serotype carbapenem-resistant hv*Kp*, was recovered from the sputum of a patient with acute bronchiolitis in Sichuan Province in November 2018 (57). The *myovirus* phage vB_KpnM_SCNJ1-Y was isolated from a river water sample and characterized in our previous study (40). It was able to form a large, clear plaque against the *K. pneumoniae* strain SCNJ1 on a Luria-Bertani (LB) agar plate. Unless otherwise specified, all bacterial strains were routinely grown at 37°C in an LB broth or LB agar. The host strain SCNJ1 was employed to amplify the phages, and the purified phage was obtained by centrifugation and filtration (0.22 µm). The phage stock solution was stored at 4°C and diluted with PBS before use.

**TABLE 2:** Bacterial strains, phages, and plasmids^*a*^

Strains or plasmids	Descriptions	References or source
*K. pneumoniae*		
SCNJ1	Clinical isolate, ST29, K54 capsule type	Laboratory stock
SCNJ2	Clinical isolate, ST11, K47 capsule type	Laboratory stock
Phage		
vB_KpnM_SCNJ1-Y	*Myovirus,* targeting the *K. pneumoniae* strain SCNJ1	(40)
*E. coli*		
DH5α	Host strain for gene cloning	Laboratory stock
BL21 (DE3)	Host strain for protein expression	Laboratory stock
Plasmid		
pSTV28-kan	Km^r^, cloning vector	Laboratory stock
pKO3-km	Km^r^, pKO3-derived plasmid	(58)
pET22b (+)	Amp^r^, His tag, protein expression vector	Laboratory stock

^*a*^ Km, kanamycin; Amp, ampicillin.

### Selection of phage-resistant clones *in vitro* and *in vivo*

#### In vitro

Three different single colonies of *K. pneumoniae* SCNJ1 were picked and then cultured separately until they reached the mid-exponential phase (the optical density at 600 nm [OD600] ~ 0.6–0.8); 1 mL bacterial culture (5×10^8^ colony-forming units [CFU]) was mixed with 10 μL purified phage suspension (5×10^7^ plaque-forming units [PFU]) at a 0.1 multiplicity of infection (MOI). The mixture was then incubated at 37°C with shaking at 220 rpm for 12 h. After incubation, 10 μL of the mixture suspension was pipetted and streaked on plates, respectively. After incubation at 37°C overnight, 20 single colonies on each plate (*n* = 3) were randomly picked and continuously purified three to five times. The phage-resistant phenotype of colonies was confirmed by inverted spotting assays. Nine representatives *in vitro* phage-resistant clones that were selected for further analysis were named YR1–YR9.

#### In vivo

Female mice aged 3–5 weeks (weighing 18–22 g) were used for this experiment. After mild isoflurane inhalation anesthesia, three mice were randomly selected, and each was intranasally inoculated with 50 μL of the SCNJ1 bacterial suspension (1 × 10⁶ CFU per mouse). In addition, one mouse was intranasally inoculated with 50 μL of PBS as a control group; 2 h later, all the mice were inoculated with 50 μL of purified phage solution (1 × 10⁸ PFU per mouse). In addition, 12 h later, all mice were euthanized, and then, their lungs were carefully removed and thoroughly ground under low temperature conditions. The lung grinding fluid was serially diluted with PBS and spread on LB plates. After incubation at 37°C overnight, 20 single colonies on each plate (n = 3) were randomly picked and continuously purified three to five times. The phage-resistant phenotype of colonies was confirmed by inverted spotting assays. Nine representatives *in vivo* phage-resistant clones that were selected for further analysis were named YM1, YM2, YM5, YM9, YM10, YM11, YM12, YM13, and YM15.

### Inverted spot plate assay

In total, 10 μL of an exponential-phase bacterial culture was separately dropped onto the double-layer agar plate containing purified phage SCNJ1-Y (10^9^~10^10^ PFU) in the top agar. After incubation at 37°C for 8–10 h, the presence or absence of a bacterial lawn indicates phage sensitivity/resistance. *K. pneumoniae* strain SCNJ1 was used as the negative control, a *K. pneumoniae* clinical isolate SCNJ2 (K47 capsular type) as the positive control, and LB was used as the blank control.

### Bacterial growth kinetics assay

Overnight culture of the phage-resistant clone or the parental strain SCNJ1 was diluted at 1:100 into fresh LB or in LB containing purified phage at MOI 0.0001; 200 μL of the bacterial cultures was separately transferred into a flat-bottomed 96-well microplate with the starting density of 1 × 10^8^ CFU/mL. The microplate was incubated at 37°C for 20 h in a microplate reader (SynergyH1, BioTek). The OD600 of the bacterial cultures was measured after continuous shaking for 3 s every 1 h, and then, all the measured values were plotted into a curve. This experiment was repeated at least three times.

### EOP assay

In total, 1 mL exponential-phase bacterial culture of phage-resistant clones or the parental strain SCNJ1 (1 × 10^9^ CFU) was mixed with 10 μL serial dilution phage suspension (10^1^–10^3^ PFU), respectively. After incubation for 10 min at 37 °C, the number of lysis plaques emerging from phage in each strain was measured by the double-layer agar method (59). EOP was calculated by dividing the average number of plaques of the test strain by the average number of plaques of the wild strain. This experiment was repeated at least three times.

### String test

The string test was performed as described previously with minor modifications (60). Briefly, bacteria were streaked onto an LB blood plate and inoculated overnight. A positive result of the string test was determined when the length of the viscous string formed by stretching a bacterial clone with a sterile loop exceeded 5 mm. Conversely, a negative result was indicated when the length of the viscous string was less than 5 mm.

### Adsorption efficiency assay

In total, 1 mL of exponential-phase bacterial cultures (1×10^8^ CFU, the test group) or sterile PBS (the control group) was mixed with 10 μL purified phage (10^4^ PFU) mixed at an MOI of 0.0001. After incubation at 37°C with shaking at 220 rpm for 10 min, the mixture was centrifuged for 5 min (12,000 rpm, 4°C). The phage titer of the supernatant was determined by the double-layer agar method, and the phage adsorbed to the host bacteria was calculated based on the titer as previously described (25). Adsorption efficiency was calculated by the following formula: ([phage titer of the control group − phage titer of the test group]/phage titer of the control group) × 100%.

### Bacterial genome sequencing and analysis

Bacterial genomic DNA was extracted using a Bacterial Genomic DNA Isolation Kit (Sangon Biotech, China) according to the manufacturer’s protocol and was sequenced on the Illumina HiSeq 2000 platform (150-bp paired reads) by Tsingke Biotech (Beijing, China). The software fastp was used to filter the raw data (61), and the resulting clean reads were aligned to the SCNJ1 reference genome (GenBank accession no. CP174529) with breseq version 0.39.0 (62). The detected mutations were subsequently manually reviewed.

### Cloning and complementation experiments

Cloning experiments were performed as previously described to investigate whether these mutations mediate phage resistance (25). The complete sequences of *wzb*, *wbbO*, *wbbN*, and *wzm* genes, including their native promoters, were amplified via PCR using specific primers (Table S3) with the genomic DNA of SCNJ1 as the template. The mutated *wbbO*^P85Q^ gene was amplified via PCR with the genomic DNA of phage-resistant mutant YR1 as the template. The cloning vector pSTV28-kan was linearized via PCR with primers pSTV28-kan-F/R (Table S3). Each purified PCR product of the target gene was ligated with pSTV28-kan by homologous recombination using the ClonExpress II One Step Cloning Kit (Vazyme, China) and chemically transformed into competent *E. coli* DH5α. The transformants were screened on LB agar plates containing 50 μg/mL kanamycin. The transformants were confirmed by PCR and Sanger sequencing using primers pSTV28sequencing-F/R. The resulting recombinant plasmid was named pSTV28-*wzb*, pSTV28-*wbbO*, pSTV28-*wbbO*^P85Q^, pSTV28-*wbbN*, and pSTV28-*wzm*, respectively. These recombinant plasmids were further separately transformed into corresponding phage-resistant mutants by electroporation. The empty pSTV-28-kan vector was electroporated into each phage-resistant mutant as a control. The phage susceptibility of these complementary strains was determined by the spot test and EOP assay.

### Spot test

In total, 200 μL exponential-phase bacterial cultures were spread on the LB agar plate, and then, 10 μL serially diluted purified phages (10^6^–10^10^ PFU/mL) were sequentially dropped onto the bacterial lawns. After overnight incubation at 37°C, the presence or absence of a cleared lysis plaque indicates phage sensitivity/resistance. LB broth was used as the negative control.

### Construction of the *ΔwbbO* mutant and its complementation strain

The generation of *K. pneumoniae* SCNJ1 Δ*wbbO* mutant was carried out as previously described (63). The left and right flanking regions of the *wbbO* gene were amplified from the chromosome of strain SCNJ1 by PCR with primers *wbbO*-L1/R1 and *wbbO*-L2/R2 (Table S3), respectively, and were ligated together via overlap extension PCR with primers *wbbO*-L1/R2. The resulting PCR product was cloned into the temperature-sensitive suicide vector pKO3-km pre-digested with NotI (New England Biolabs) by homologous recombination with the ClonExpress II One Step Cloning Kit (Vazyme, China). The resulting recombinant plasmid was electroporated into *K. pneumoniae* strain SCNJ1 and plated on LB agar plates containing kanamycin (50 μg/mL) at the non-permissive temperature (43°C) to force integration of the plasmid into the bacterial chromosome. Correct allelic exchange was verified by PCR using primers pKO3-km-F/R and primers (*wbbO*-L1 long/R2 long) flanking the *wbbO* gene. Three colonies from the first round of DNA exchange were picked, serially diluted, and plated at 30°C on 10% sucrose plates. After incubation for ~24 h, kanamycin-sensitive colonies were picked, and the deletion of *wbbO* was confirmed by PCR and Sanger sequencing using primers *wbbO*-L1 long/R2 long. For the complementation, the recombinant plasmids pSTV28-*wbbO* and pSTV28-*wbbO*^P85Q^ were electroporated into the Δ*wbbO* mutant, respectively, yielding the recombinant strains Δ*wbbO/*pSTV28-*wbbO* and Δ*wbbO/*pSTV28-*wbbO*^P85Q^.

### Extraction and quantification of CPS and LPS

LPS was extracted using the LPS extraction kit (Bestbio, China) according to the manufacturer’s protocol and quantified by the phenol-sulfuric acid method (64). The extracted LPS samples were placed in a freeze vacuum dryer (LABCONCO, USA) for drying and concentration for 24 h. The lyophilized product was dissolved in sterile ddH_2_O before use.

Uronic acid is an essential constituent and a biomarker of the capsule (41). Capsule biomass of the strains was extracted and quantified by uronic acid assay as previously described (63, 65). The uronic acid was extracted from an overnight bacterial culture with Zwittergent 3–14 in 100 mM citric acid. The mixture was then precipitated by adding absolute ethanol and resuspended in the tetraborate/sulfuric acid solution and then boiled for 5 min. After that, 3-hydroxy diphenol in 0.5% NaOH was added, and the absorbance was measured at 520 nm to determine the amount of uronic acid. A standard curve was generated with glucuronic acid.

### Phage infection inhibition assay

Equal volumes of pre-warmed (37°C) phage samples (10^4^ PFU) and LPS (100 μg/mL) or CPS (100 μg/mL) were mixed and incubated at 37°C (28). Aliquots were taken after 90 min of incubation and titrated by the double-layer agar method. Infection efficiency was calculated by dividing the average number of plaques of the test group by the average number of plaques of the control group (ddH_2_O).

### Protein expression

The depolymerase gene *dep_Y* and tail fiber gene *tfp_Y* were amplified from the genomic DNA of phage SCNJ1-Y using primers Dep-F/R and TFP_Y-F/R by PCR, respectively. The purified PCR product was double-digested with BamHI and XhoI restriction endonucleases (New England Biolabs) according to the manufacturer’s protocol. The digested product was then ligated into the expression vector pET22b (+), which had been pre-digested with the same enzymes (BamHI and XhoI), using T4 DNA ligase (New England Biolabs). The recombinant plasmid was chemically transformed into *E. coli* BL21 (DE3), and the transformants were screened on LB agar plates containing 100 μg/mL ampicillin. The successful construction of the recombinant plasmid was confirmed by PCR and Sanger sequencing using universal primer of pET22b(+). *E. coli* BL21(DE3) cells transformed with recombinant plasmid were grown in LB broth containing ampicillin (100 μg/mL) to an exponential phase (OD600 ~0.6), and the protein expression was induced with 0.5 mM isopropyl-β-D-thiogalactopyranoside (IPTG) for 12 h at 25°C. Cells were harvested by centrifugation at 8,000 rpm for 10 min (4°C) and resuspended in ice-cold phosphate-buffered saline (PBS, pH 7.4). The cells were lysed by sonication with 5 s pulse/3 s interval cycles. After centrifugation, the supernatant was filtered through a 0.22 µm filter. Protein with a 6×His tag was purified using a 1 ml HisTrap Ni-NTA column (Yeasen, China) according to the manufacturer’s instructions. The purified recombinant proteins Dep_Y and TFP_Y were analyzed using 12% sodium dodecyl sulfate-polyacrylamide gel electrophoresis (SDS-PAGE) and Coomassie Brilliant Blue staining. The concentration of recombinant protein was determined by Bradford assay (66).

### Capsule degradation assay and Alcian blue staining

In total, 8 μg CPS was incubated with 3 μg Dep-Y or an equal volume of ddH_2_O for 2 h at 37°C. Following the incubation, samples were checked by Alcian blue staining after 8% SDS-PAGE separation and visualized by Alcian blue staining as previously described (38, 67). Briefly, the gel was washed three times (10 min each) with fixing buffer (25% ethanol, 10% acetic acid) at 55°C. After that, the gel was stained with 0.125% Alcian blue (Macklin, China) in fixing buffer for 10 to 15 min at 55°C in the dark, and then destained with fixing buffer overnight.

### Biofilm formation

The biofilm formation assays were performed as previously described with minor modifications (63, 68). Overnight bacterial culture was diluted at 1:100 into 200 µL fresh LB broth and inoculated statically in technical triplicate in sterile polystyrene round-bottom 96-well microtiter plates (Corning, USA). After incubation for 12 h, 24 h, or 48 h at 37°C, the supernatant was removed, and the wells were washed with water by immersion and gentle shaking. After fixing with methanol for 10 min, the biofilm was stained with 1% crystal violet (Solarbio, China) for 15 min. Crystal violet solution was removed, and the biofilm was washed with ddH_2_O three times. The bound dye was released by adding 200 µL of acetic acid (33%, vol/vol), and absorbance was measured at 595 nm using a microplate reader (BioTek Synergy H1, USA). The experiment was repeated at least three times.

### Human serum killing assay

The serum bactericidal assay was performed as described previously with minor modifications (69, 70); 4 × 10^6^ CFU of exponentially growing bacteria were suspended in PBS and mixed with pooled human serum from healthy volunteers at a 1:3 vol/vol ratio in a final volume of 400 µL. The mixture was incubated at 37 °C for 3 h without shaking. At the time points of 0, 1, 2, and 3 h, 100 μL aliquots were retrieved, diluted, and plated on LB agar plates to determine the number of viable bacteria. This experiment was repeated at least three times in triplicate.

### Mouse experiment

Female BALB/c mice aged 6–8 weeks with a weight of 18–22 g were raised under standard experimental conditions and allowed food and water *ad libitum*.

#### Survival assay

The mice were randomly divided into different groups, with 10 mice in each group. After being mildly anesthetized with isoflurane, mice were intranasally infected with 50 μL of bacterial suspension in PBS (SCNJ1, YR1, YR7, YM11, or YM13, 2 × 10^7^ CFU per mouse). In addition, three mice were inoculated with 50 μL of PBS as a control. The survival status of the mice was recorded every 12 h for 7 consecutive days, with an in extremis state or death as the study endpoint.

#### Organ burden assays and histopathology

The mice were randomly divided into different groups, with 5 mice in each group. After being mildly anesthetized with isoflurane, mice were intranasally infected with 50 μL of bacterial suspension (SCNJ1, YR1, YR7, YM11, or YM13, 1 × 10^6^ CFU per mouse). Three mice were inoculated with 50 μL of PBS as a control; 48 h after infection, all the mice were euthanized, and their lungs, livers, and spleens were carefully removed. The left lungs of the mice were fixed with 1 mL of 10% neutral buffered formalin for 24 h. Then, the lung tissues were embedded, sliced, and stained with hematoxylin-eosin (HE). The livers, spleens, and right lungs of the mice were weighed under sterile conditions, respectively, and then, each tissue was ground with 1 mL of PBS at a low temperature. After serial dilution, the homogenates were spread on LB agar plates containing 100 μg/mL ampicillin and incubated at 37°C overnight for colony counting. The organ burden was standardized with the number of bacteria per 1 g wet organ weight.

### DNA ejection assay

DNA ejection assay was performed as described previously with minor modifications (46); 500 μL of phage particles (8.89 × 10^10^ PFU/mL) were mixed with 10 μL of LPS (0.2 mg/mL) of strain SCNJ1 (named SCNJ1-LPS), and then incubated overnight at 37 °C. In total, 5 units of DNase I were added at the end of ejection as a control for DNA accessibility. The released DNA of the phage particles was detected on a 1.2% agarose gel electrophoresis followed by staining with ethidium bromide, and the results were observed using a gel imaging system (Analytik Jena, Germany). For the DNA ejection inhibition assay, 500 μL of phage particles (8.89 × 10^10^ PFU/mL) were mixed with 10 μL of SCNJ1-LPS (0.2 mg/mL). Then, 5 μL TFP_Y (300 μg/mL, suspended in 50 mM imidazole solution) was added to the mixture. An equal volume of 50 mM imidazole solution was used as a control. After incubation at 37 °C for 6 h, the product was detected using the 1.2% agarose gel electrophoresis along with ethidium bromide staining.

### Isolation of mutant phage and phage genome sequencing

Isolation of the mutant phage was followed by a previously described method with minor adjustments (71); 10 μL serially diluted purified phages (10^4^–10^11^ PFU/mL) were sequentially dropped onto the double-layer agar plate containing 100 μL YR1 (an LPS-related phage-resistant clone) bacterial culture (OD600~ 2.5) in the top agar. LB broth was used as the negative control. After overnight incubation at 28°C, the most cleared lysis plaque was picked for phage amplification at 28°C overnight. The above process was repeated at least three times until the spot was completely transparent. The resulting lysis plaque was subjected to three rounds of purification by plaque picking and passaging using the double-layer agar method. This experiment was carried out in triplicate.

The genome DNA of the mutant phages was extracted from a high-titer phage stock using the Viral DNA kit (omega BIO-TE, USA) following the manufacturer’s instructions. The purified DNA was sequenced on the Illumina HiSeq 2000 platform (150-bp paired reads) by Tsingke Biotech (Beijing, China). The raw data were filtered using the software fastp (61), and the resulting clean reads were aligned to the genome sequences of phage SCNJ1-Y (GenBank accession no. OQ689083) using breseq version 0.39.0 (62).

### Bioinformatics analyses and molecular docking

BLASTp analysis (https://blast.ncbi.nlm.nih.gov, accessed on 17 June 2025) was performed using the NCBI website platform with default parameters. Protein homology detection was conducted on the Swiss-Model server using the primary sequences as the search hint with default values (72). EMBOSS Needle (https://www.ebi.ac.uk/jdispatcher/psa/emboss_needle) was used to align amino acid sequences to identify regions of similarity and conservation. AlphaFold was used to predict the three-dimensional structure of TFP_Y (73). The predicted protein structure was then used as a query to search against the DALI server’s database of known protein structures (74). Espript 3.0 (75) was used to visualize the alignment result, and structure figures were visualized using Chimera (76).

### Statistical analysis

The data in this study were shown in the form of mean ± standard deviation (SD), and data analyses were performed using GraphPad Prism 10.1.2. The phage adsorption efficiency, phage infection inhibition assay, and organ burden assays were analyzed using one-way ANOVA with Dunnett’s post hoc test. The biofilm formation and serum resistance assays were analyzed using two-way ANOVA, and the survival curves were analyzed using the log-rank (Mantel-Cox) test. Differences were judged statistically significant at a *P* value of < 0.05.

## Data Availability

The Illumina raw reads of phage-resistant mutants and mutant phages are available in the NCBI SRA database under bioprojects PRJNA1261817 and PRJNA1262362, respectively. Other data and materials are available from the corresponding authors on reasonable request.

## SUPPLEMENTAL MATERIAL

Tables S1 to S3 and Figures S1 to S7 spectrum.02171-25-s0001.docx Table S1: The EOP assays of representative mutants and their complementation strain. Table S2: SNPs in the mutant phages. Table S3: Primers used in this study. Fig. S1: Growth curves of phage-resistant mutants. Fig. S2: Colony morphology and mucoid phenotype. Fig. S3: EOP assay of SCNJ1, YR7, and YR7/pstv28-wzb. Fig. S4: Growth curves of SCNJ1, YR7, and YR7/pstv28-wzb. Fig. S5: EOP assay of LPS-related mutants and the complementation stains. Fig S6: Growth curves of LPS-related mutants and the complementation stains. Fig S7: Circular diagram of the genome of phage SCNJ1-Y.

ASM does not own the copyrights to Supplemental Material that may be linked to, or accessed through, an article. The authors have granted ASM a non-exclusive, world-wide license to publish the Supplemental Material files. Please contact the corresponding author directly for reuse.
