# Supplementary material for: A novel tail fiber protein triggers phage DNA ejection by recognizing lipopolysaccharides of K54 hypervirulent Klebsiella pneumoniae
Source: Microbiol Spectr. 2025 Nov 10;13(12):e02171-25. doi: 10.1128/spectrum.02171-25 (PMC12671077; doi:10.1128/spectrum.02171-25)
Supplement: Tables S1 to S3 and Figures S1 to S7 — Table S1: The EOP assays of representative mutants and their complementation strain. Table S2: SNPs in the mutant phages. Table S3: Primers used in this study. Fig. S1: Growth curves of phage-resistant mutants. Fig. S2: Colony morphology and mucoid phenotype. Fig. S3: EOP assay of SCNJ1, YR7, and YR7/pstv28-wzb. Fig. S4: Growth curves of SCNJ1, YR7, and YR7/pstv28-wzb. Fig. S5: EOP assay of LPS-related mutants and the complementation stains. Fig S6: Growth curves of LPS-related mutants and the complementation stains. Fig S7: Circular diagram of the genome of phage SCNJ1-Y. [file spectrum.02171-25-s0001.docx]

***Supplementary files***

**A novel tail fiber protein triggers phage DNA ejection by recognizing lipopolysaccharides of K54 hypervirulent *Klebsiella pneumoniae***

Ming Yin^1,^ ^†^ , Li Cao^1, †^, Yu Fu^1^^,^ ^†^, Yanjun Lu^1^, Yi Yan^1^, Lvxin Qian^1^, Li Xiang^1^, Tiejun Zhou^2^, Huan Chen^1^, Ying Li^1,*^, Luhua Zhang^1,*^

1. The School of Basic Medical Sciences, Southwest Medical University, Luzhou, Sichuan, China
2. Department of Pathology, the Affiliated Hospital of Southwest Medical University, Luzhou, Sichuan, China

*Correspondence: Luhua Zhang, [zhluhua@swmu.edu.cn](mailto:zhluhua@swmu.edu.cn) or Ying Li, [lying1019@swmu.edu.cn](mailto:lying1019@swmu.edu.cn).

**^†^** These authors contributed equally: Ming Yin, Li Cao, and Yu Fu.

**Table S1 The EOP assays** **of representative mutants and their complementation strains.**

| **Strain name** | **EOP value (%)** |
| --- | --- |
| SCNJ1 | 100 |
| YR1 | 0 |
| YR1/pSTV28 | 0 |
| YR1/pSTV28-*wbbO* | 86.85±2.68 |
| YR7 | 0 |
| YR7/pSTV28 | 0 |
| YR7/pSTV28-*wzb* | 95.02±0.37 |
| YM11 | 0 |
| YM11//pSTV28 | 0 |
| YM11//pSTV28-*wzm* | 97.69±4.80 |
| YM13 | 0 |
| YM13/pSTV28 | 0 |
| YM13/pSTV28-*wbbN* | 82.68±4.93 |
| Δ*wbbO* | 0 |
| Δ*wbbO*/pSTV28-*wbbO*^WT^ | 28.41±2.51 |
| Δ*wbbO*/pSTV28-*wbbO*^P85Q^ | 0 |

**Table S2** **SNPs in the mutant phages.**

| **Isolate** | **Position**  **(bp)** | **Gene** | **Annotation** | **Putative gene function** |
| --- | --- | --- | --- | --- |
| SCNJ1-Y_mut1 | 23,570 | *tfp*1 | G195D (GGT→GAT) | Tail fiber protein |
|  | **29,882** | ***tfp_Y*** | **T169I (ACA→ATA)** | **Tail fiber protein** |
|  | **30,001** | ***tfp_Y*** | **S209G (AGT→GGT)** | **Tail fiber protein** |
| SCNJ1-Y_mut2 | **29,882** | ***tfp_Y*** | **T169I (ACA→ATA)** | **Tail fiber protein** |
|  | **30,001** | ***tfp_Y*** | **S209G (AGT→GGT)** | **Tail fiber protein** |
| SCNJ1-Y_mut3 | 29,025 | *gp041* | Q122H (CAG→CAC) | Structural protein |
|  | **29,882** | ***tfp_Y*** | **T169I (ACA→ATA)** | **Tail fiber protein** |
|  | **30,001** | ***tfp_Y*** | **S209G (AGT→GGT)** | **Tail fiber protein** |

**Table S3 Primers used in this study.**

| **Primer** | **Sequence (5'→3')** |
| --- | --- |
| pSTV28-*kan*-F | GCTTATCGATGATAAGCTGTCAAACA |
| pSTV28-*kan*-R | AGCTGTTTCCTGTGTGAAATTGTT |
| pSTV28sequencing-F | CACAGGAAACAGCTATG |
| pSTV28sequencing-R | GCTTATCATCGATAAGC |
| *kan*-F | CACCGAGGCAGTTCCATA |
| *kan*-R | CTCTTCCGACCATCAAGC |
| *wbbO*-F | atttcacacaggaaacagctATGAGAAAATTGTGTTATTTCATAAATTCG |
| *wbbO*-R | acagcttatcatcgataagcTCATCGAACTACATCATGATATATTTGC |
| *wzb*-F | atttcacacaggaaacagctATGAAGAAAAAAATTGTTAGATTTTCG |
| *wzb*-R | acagcttatcatcgataagcTCACCAAGTTCGAATATATTTACCTGT |
| *wbbN*-F | atttcacacaggaaacagctATGAAATATACGGCATTGATAGTGACA |
| *wbbN*-R | acagcttatcatcgataagcTTAATGATACTTACCACTAATACCTTTTATGC |
| *wzm*-F | atttcacacaggaaacagctATGAAGTACAATTTAGGGTATTTATTTGATT |
| *wzm*-R | acagcttatcatcgataagcTTACAAGATCTCTGCAAATCGATATTT |
| *wbbO*-L1 | cgactctagaggatcgcggccgcTACGATGACGATGCTTACC |
| *wbbO*-L2 | CTATCACAACCACCAACATCATGGCTGATGATGTGAATCT |
| *wbbO*-R1 | AGATTCACATCATCAGCCATGATGTTGGTGGTTGTGATAG |
| *wbbO*-R2 | cggtacccggggatcgcggccgcTGGCATTGGAGCATACTG |
| *wbbO*-L1long | ATACGGCATTGATAGTGACA |
| *wbbO*-R2long | TCGGCACTAAGCATAGATG |
| pKO3-km-F | GCGATCCTCTAGAGTCGACCTG |
| pKO3-km-R | GCGATCCCCGGGTACCGAGAC |
| Dep-F | CGCggatccGATGACCATTATCAAACGCGC |
| Dep-R | CCGctcgagAACAGCCGTACCAGTAGCATCA |
| TFP_Y-F | CGCggatccGatggctttagaccgtagcaac |
| TFP_Y-R | CCGctcgagtgagtattcttcgatgataacgat |


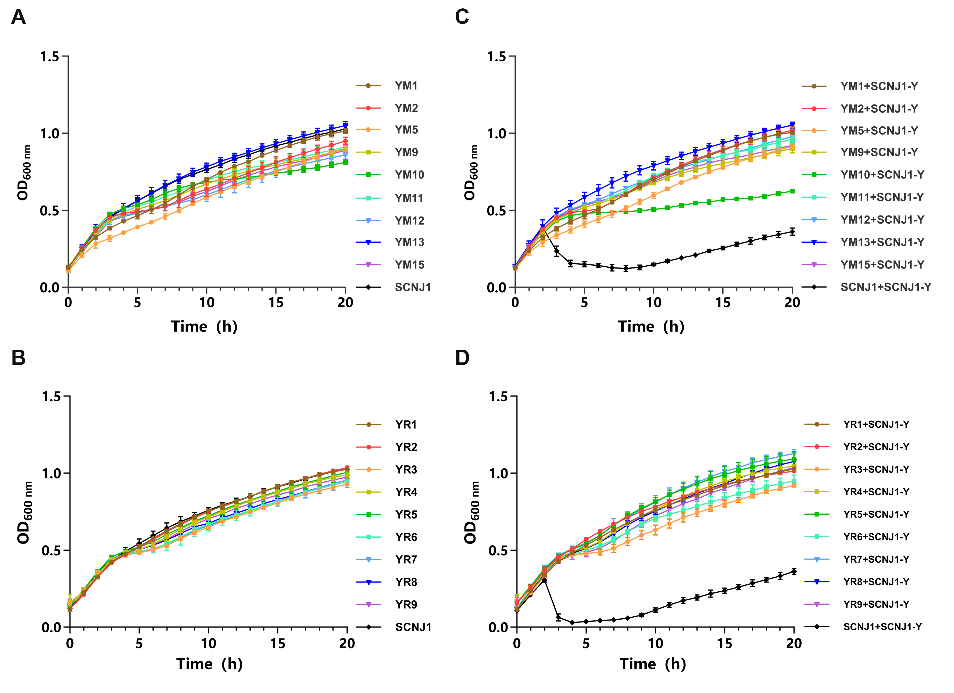


**Fig S1** **Growth curves of phage-resistant mutants.** (A)、(B) The growth curves of the wild-type SCNJ1 and phage-resistant mutants in LB medium. (C)、(D) The growth inhibition curves of phage against SCNJ1 and phage-resistant mutants at MOI of 0.0001. The data are shown as mean ± SD (n=3) from a representative experiment. OD600 nm, optical density at 600 nm.


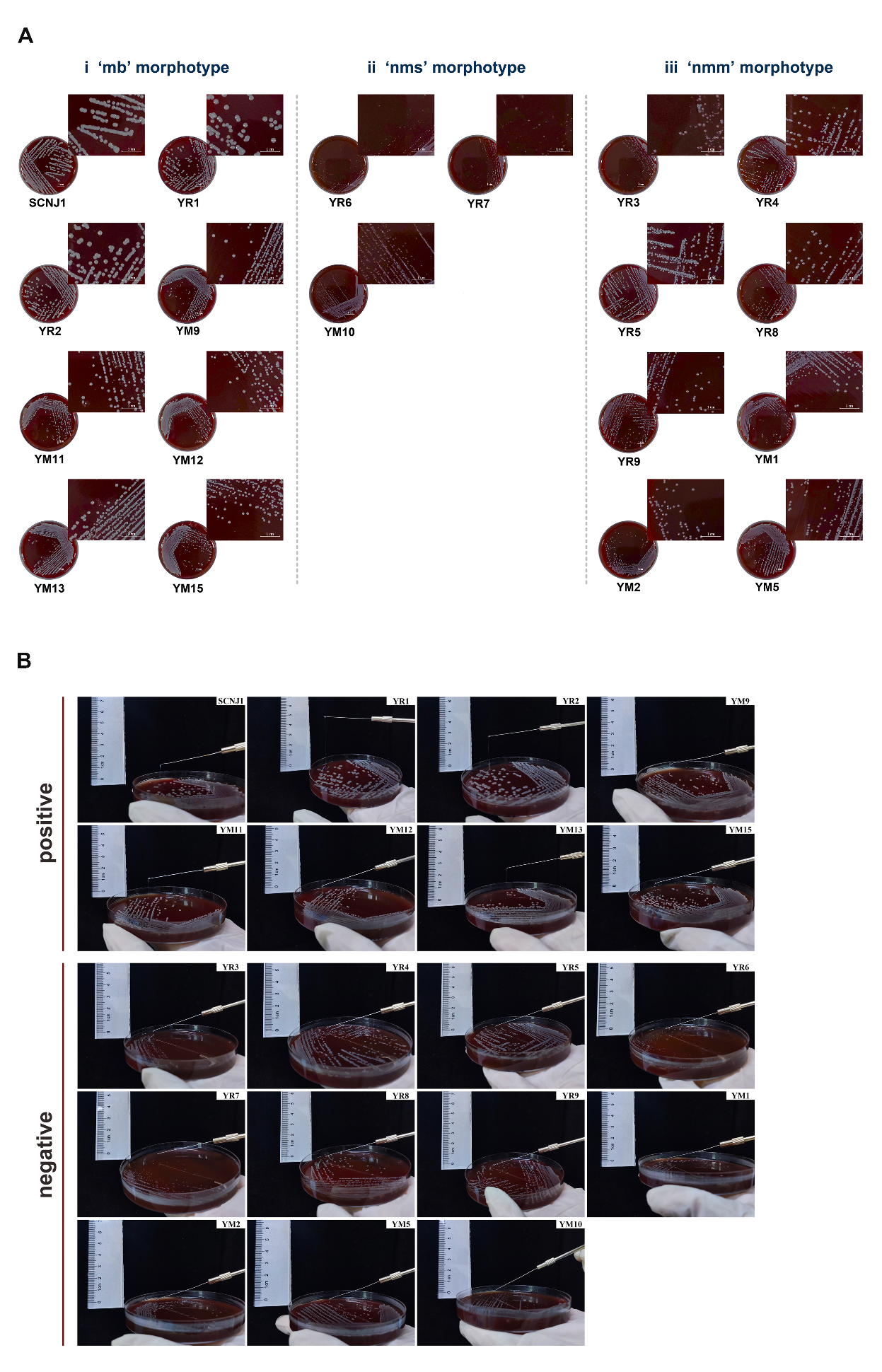


**Fig S2 Colony morphology and mucoid phenotype.** (A) Colony appearances of phage-resistant clones on LB blood agar plates. Boxes indicate areas of increased magnification. (B) The string test. The viscous strings from colonies of YR1,YR2, YM9, YM11, YM12, YM13, and YM15 exceed 5 mm in length, indicating a positive string test.


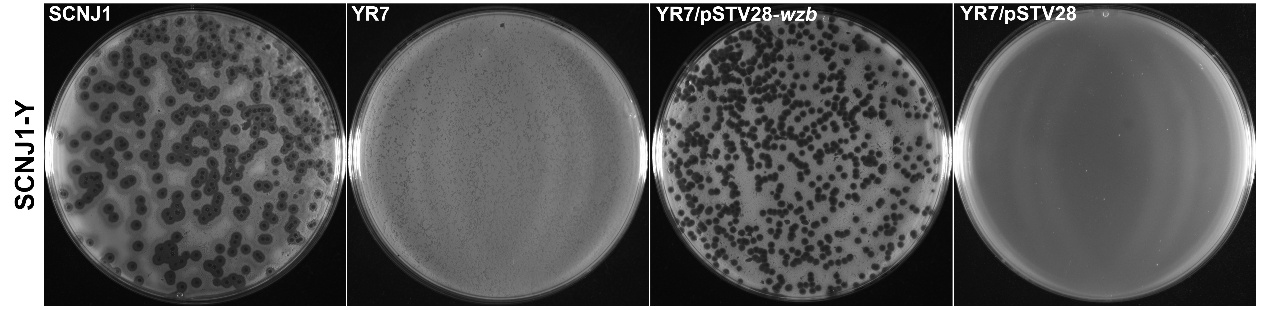


**Fig S3** **EOP assay of SCNJ1, YR7, and YR7/pstv28-*wzb*.** Phage was inoculated with bacterial culture and the number of lysis plaques in each strain was measured by the double-layer agar method.


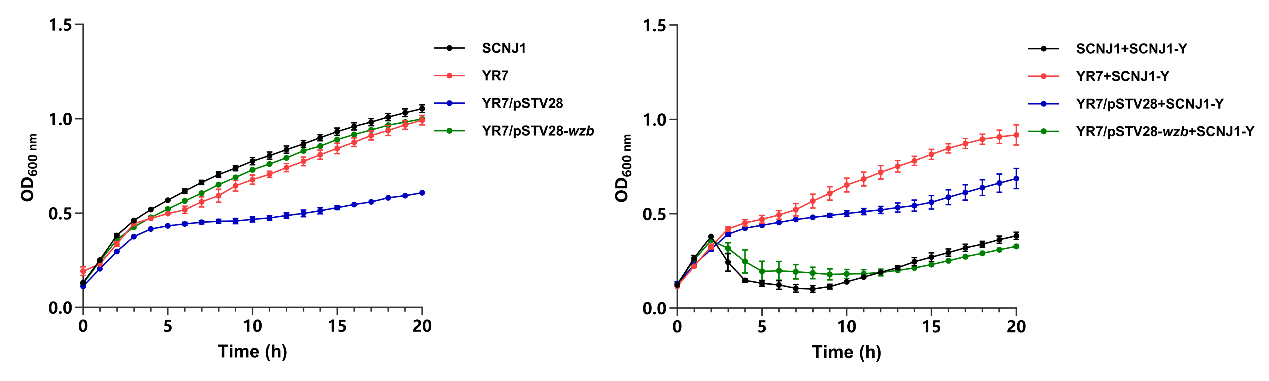


**Fig S4 Growth curves of SCNJ1, YR7, and YR7/pstv28-*wzb*.** (A) The growth curves in LB medium. (B) The growth inhibition curves of phage against SCNJ1, YR7, and YR7/pstv28-*wzb* at MOI of 0.0001. The data are shown as mean ± SD (n=3) from a representative experiment.


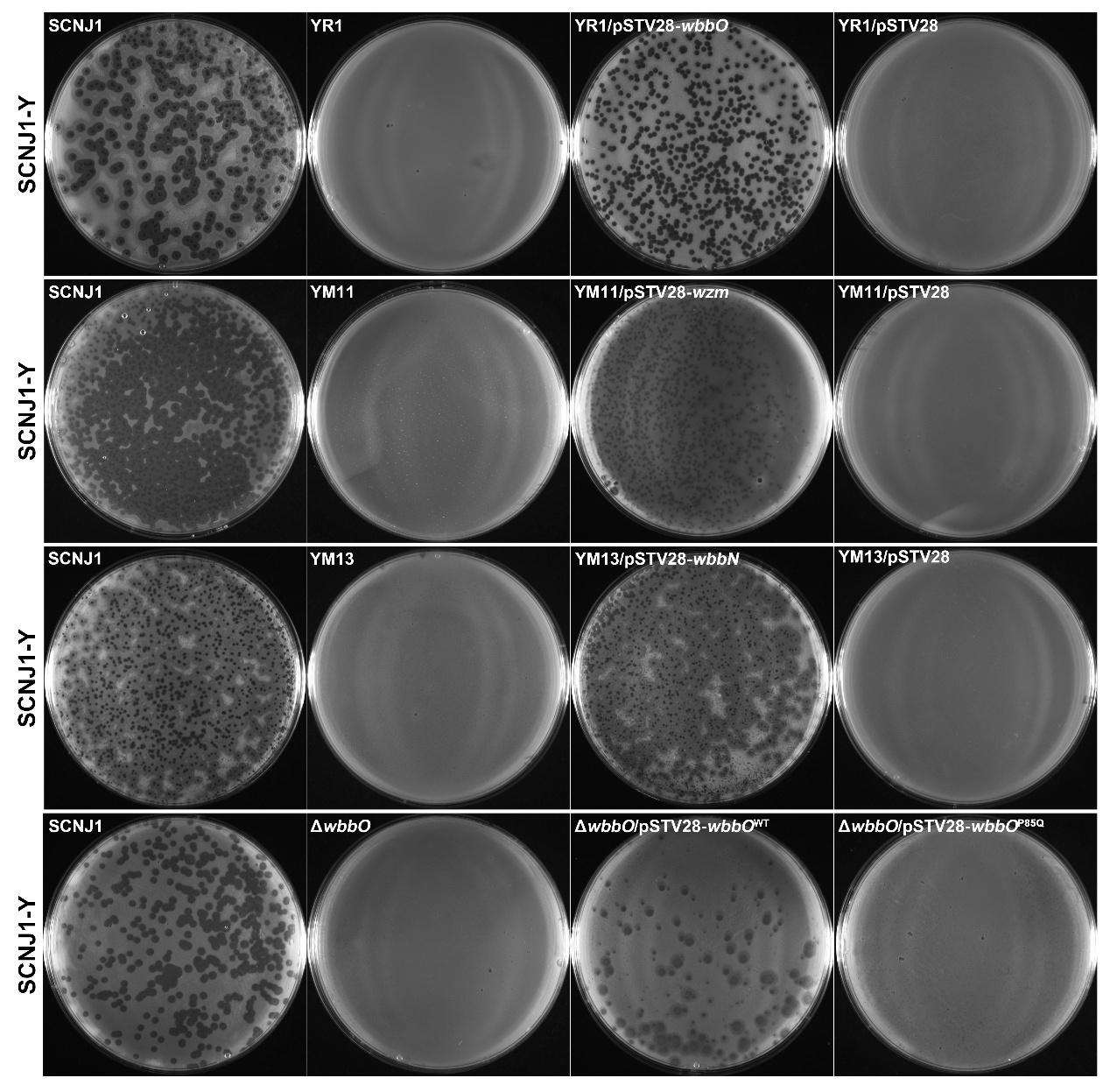


**Fig S5** **EOP assay of LPS-related mutants and the complementation stains.** Phage was inoculated with bacterial culture and the number of lysis plaques in each strain was measured by the double-layer agar method.


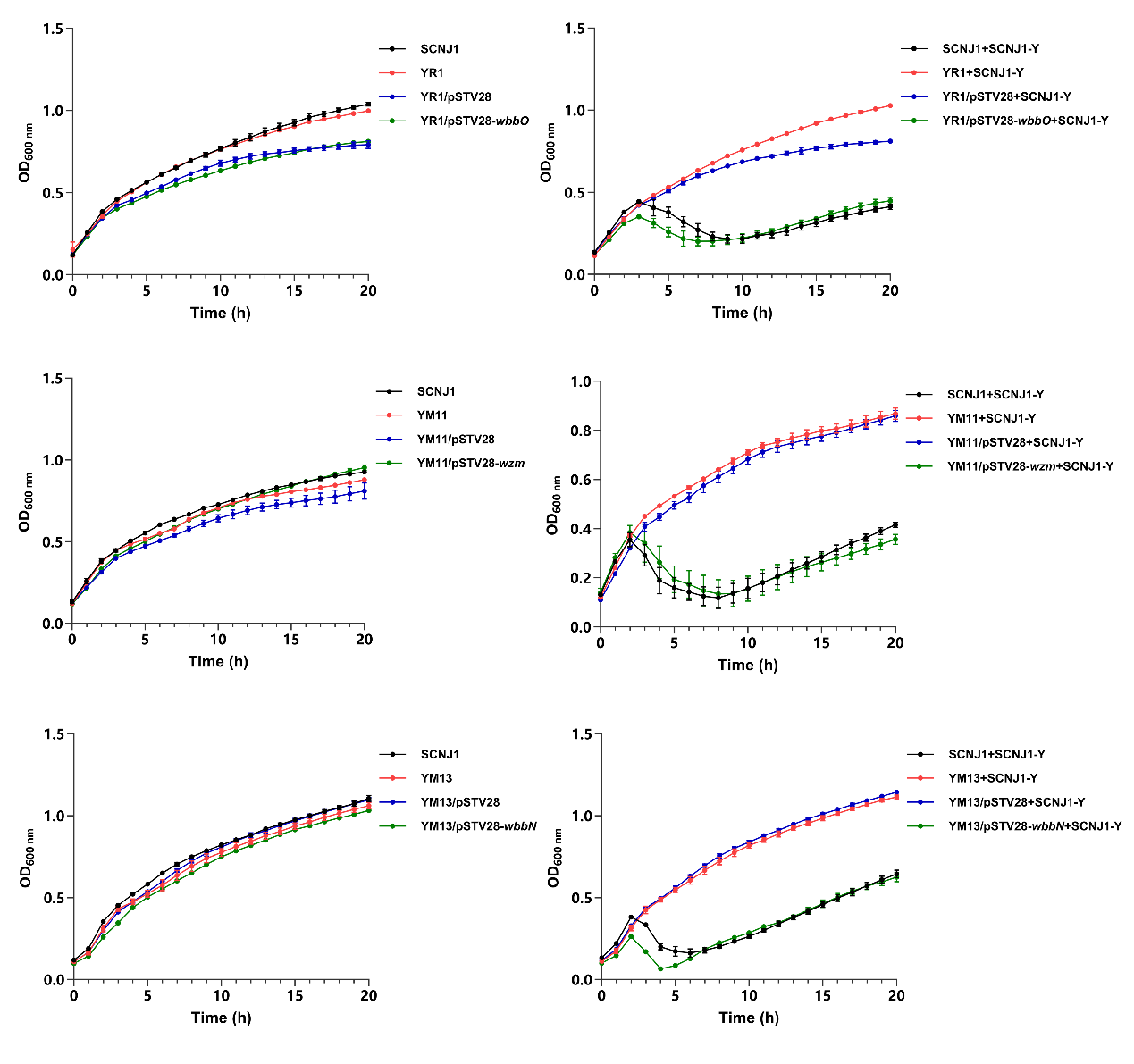


**Fig S6** **Growth curves of LPS-related mutants and the complementation stains.** (A) The growth curves in LB medium. (B) The growth inhibition curves of phage against bacterial strains at MOI of 0.0001. The data are shown as mean ± SD (n=3) from a representative experiment.


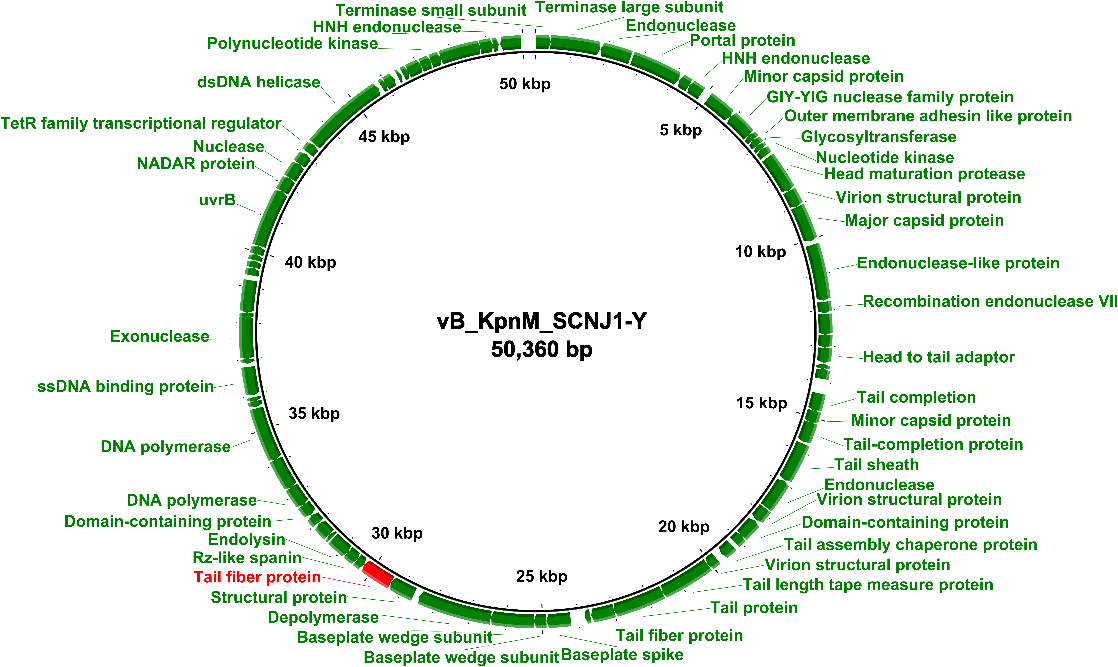


**Fig S7** **Circular diagram of the genome of phage SCNJ1-Y.** Arrows on the outer ring indicate deduced ORFs and their orientations. The mutated tail fiber protein is highlighted in red. The genome map was generated using BRIG ^1^.

1. Alikhan NF, Petty NK, Ben Zakour NL, Beatson SA. BLAST Ring Image Generator (BRIG): simple prokaryote genome comparisons. *BMC Genomics* 2011;12:402.
